# Adipose group 1 innate lymphoid cells promote adipose tissue fibrosis and diabetes in obesity

**DOI:** 10.1038/s41467-019-11270-1

**Published:** 2019-07-22

**Authors:** Hongdong Wang, Lei Shen, Xitai Sun, Fangcen Liu, Wenhuan Feng, Chunping Jiang, Xuehui Chu, Xiao Ye, Can Jiang, Yan Wang, Pengzi Zhang, Mengwei Zang, Dalong Zhu, Yan Bi

**Affiliations:** 1 Department of Endocrinology, Drum Tower Hospital Affiliated to Nanjing University Medical School, Nanjing, 210008 China; 2 Shanghai Institute of Immunology, Shanghai Jiao Tong University School of Medicine, Shanghai, 200025 China; 3 Department of General Surgery, Drum Tower Hospital Affiliated to Nanjing University Medical School, Nanjing, 210008 China; 4 Drum Tower Hospital Clinical College of Nanjing Medical University, Nanjing, 210008 China; 5 Department of Molecular Medicine, Barshop Institute for Longevity and Aging Studies, University of Texas Health San Antonio, San Antonio, TX 78229-3900 USA

**Keywords:** Innate immune cells, Diabetes

## Abstract

Pathogenic factors driving obesity to type 2 diabetes (T2D) are not fully understood. Group 1 innate lymphoid cells (ILC1s) are effectors of innate immunity and enriched in inflamed tissues. Here we show that the number of adipose ILC1s increases in obese T2D patients and correlates with glycemic parameters and with the number of ILC1s in the blood; circulating ILC1 numbers decrease as a result of metabolic improvements after bariatric surgery. In vitro co-culture experiments show that human adipose ILC1s promote adipose fibrogenesis and CD11c^+^ macrophage activation. Reconstruction of the adipose ILC1 population in *Prkdc*^*−/−*^*IL2rg*^*−/−*^ mice by adoptive transfer drives adipose fibrogenesis through activation of TGFβ1 signaling; however, transfer of *Ifng*^*−/−*^ ILC1s has no effect on adipose fibrogenesis. Furthermore, inhibiting adipose accumulation of ILC1s using IL-12 neutralizing antibodies attenuates adipose tissue fibrosis and improves glycemic tolerance. Our data present insights into the mechanisms of local immune disturbances in obesity-related T2D.

## Abstract (web summary)

Whether adipose ILC1s regulate adipose tissue fibrogenesis is unknown. Here, the authors report a direct role of adipose ILC1s in adipose tissue fibrogenesis and provide insights into the mechanisms of local immune dysfunction in obesity-associated metabolic disorders.

## Introduction

Type 2 diabetes (T2D), one of the largest public health challenges worldwide, is related to the dramatically increased incidence of obesity^1,2^, with ~60–90% of patients with T2D being obese^3,4^. Emerging findings indicate that innate and adaptive immune responses in adipose tissue have critical functions in the regulation of metabolic homeostasis, while in obesity, type 1 inflammation-associated immune cells are predominant in adipose tissue and exert metabolically deleterious impacts^5^. Clarifying the precise alteration of the adipose tissue immune system and subsequent effect on metabolic homeostasis is urgently needed to counter the rise in obesity-associated T2D.

Innate lymphoid cells (ILCs) are effectors and regulators of innate immunity, and can be found in both lymphoid and non-lymphoid peripheral tissues^6^. On the basis of the expression of transcription factors, cell surface markers, and effector cytokines, mature ILCs can be classified into groups 1, 2, or 3 ILCs^6^, of which, group 1 ILCs include conventional natural killer (cNK) cells and subsets of unconventional NK cells^7^. During the development of group 1 ILCs, key transcription factors TOX, NFIL3, and Id2 are required at an early stage of differentiation, whereas the master transcription factor, T-bet, is important for the interferon (IFN)-γ-producing function^6,8^. Interestingly, ILC1s were previously reported to be particularly prominent under inflammatory conditions in mucosal tissues from Crohn’s disease patients^9,10^, indicating the role for ILC1s in the pathogenesis of gut mucosal inflammation. A study has shown that accumulation of adipose ILC1s directly induces local inflammation and systemic insulin resistance in high-fat-diet (HFD)-fed mice^11^. However, whether adipose ILC1s contributes to the development of T2D in obese individuals is not clear.

Adipose tissue fibrosis in obese individuals, caused by pathologically excessive accumulation of the extracellular matrix (ECM), is a hallmark of malfunction that is linked to insulin resistance and T2D^12–14^, while repression of adipose tissue fibrosis improves glucose homeostasis^15^. Adipose tissue macrophages (ATMs) are reported to have an important function in regulating adipose tissue fibrosis: macrophage-secreted factors are shown to promote a profibrotic phenotype in preadipocytes;^16^ in addition, proinflammatory CD11c^+^ macrophages drive adipose tissue fibrogenesis by regulating distinct cell types^17,18^. Adipose ILC1s were reported to promote CD11c^+^ macrophage activation in HFD-induced mice^11^, but whether adipose ILC1s regulate adipose tissue fibrogenesis and the underlying mechanisms are unclear.

Here, we show that the number of adipose ILC1s increases in obese T2D patients and induces adipose fibrogenesis in an IFN-γ-dependent fashion. Reconstruction of adipose ILC1s in *Prkdc*^*−/−*^*IL2rg*^*−/−*^ mice drives adipose fibrogenesis through activation of transforming growth factor β-1 (TGF-β1) signaling, whereas inhibiting adipose ILC1s accumulation attenuates adipose tissue fibrosis and improves glycemic intolerance. Thus, our data present mechanistic insights into local immune disturbances in obesity-associated T2D.

## Results

### Adipose ILC1s correlate with the development of obesity-associated T2D

To evaluate the role of adipose ILC1s in the development of obese T2D, we enrolled control subjects (*n* = 36), obese individuals (*n* = 27), and obese T2D patients (*n* = 22), whose clinical and biochemical characteristics of the enrolled subjects are summarized in Table 1. There were no differences in age and sex between controls, obese subjects, and obese T2D patients.

**Table 1:** Characteristics of subjects whose blood samples were analyzed

	Control	Obese	Obese T2D	*P-*values			
				Overall	Control vs. obese	Control vs. obese T2D	Obese vs. obese T2D
*N*	36	27	22	–	–	–	–
Age (years)	42.6 ± 6.5	40.0 ± 13.6	38.7 ± 11.2	0.353	–	–	–
Male, *n* (%)	8 (22.2)	8 (29.6)	6 (27.3)	0.796	–	–	–
BMI (kg m^−2^)	23.4 ± 2.8	35.4 ± 7.1	38.9 ± 5.3	0.000	0.000	0.000	0.020
HbA1c (%)	5.4 ± 0.4	5.7 ± 0.5	8.4 ± 1.0	0.000	0.149	0.000	0.000
Fasting glucose (mmol l^−1^)	4.8 ± 0.4	5.4 ± 0.9	11.0 ± 3.5	0.000	0.189	0.000	0.000
2 h post (mmol l^−1^)^a^	5.7 ± 0.9^a^	7.1 ± 1.5	16.7 ± 4.1	0.000	0.309	0.000	0.000
Fasting insulin (mIU ml^−1^)	7.0 ± 8.6	22.5 ± 14.2	31.5 ± 19.9	0.000	0.000	0.000	0.027
HOMA-IR (units)	1.5 ± 2.0	5.7 ± 4.0	14.8 ± 9.1	0.000	0.003	0.000	0.000
Triglycerides (mmol l^−1^)	1.2 ± 0.5	1.8 ± 0.8	4.2 ± 3.5	0.000	0.017	0.000	0.000
Total cholesterol (mmol l^−1^)	4.6 ± 0.9	4.5 ± 0.9	5.1 ± 1.1	0.071	–	–	–
HDL-C (mmol l^−1^)	1.4 ± 0.4	1.1 ± 0.3	1.0 ± 0.1	0.000	0.001	0.000	0.140
LDL-C (mmol l^−1^)	2.7 ± 0.7	2.8 ± 0.7	2.6 ± 0.6	0.738	–	–	–
Fasting FFA (mmol l^−1^)	0.4 ± 0.2	0.6 ± 0.1	0.7 ± 0.1	0.000	0.000	0.000	0.117
Adipo-IR (mIU ml^−1^ × mmol l^−1^)	2.8 ± 3.6	15.0 ± 10.7	22.4 ± 14.7	0.000	0.000	0.000	0.010

*BMI* body mass index, *FFA* free fatty acid*, HOMA-IR* homeostasis model assessment-insulin resistance, *HDL-C* high-density lipoprotein cholesterol, *LDL-C* low-density lipoprotein cholesterol, *Adipo-IR* adipose insulin resistance index

All data are presented as mean  ±  SD or *n* (%). Comparisons are by ANOVA and, when appropriate, LSD post hoc test or χ^2^ test

^a^The data of 25 non-obese control subjects were missed

**Fig. 1:**
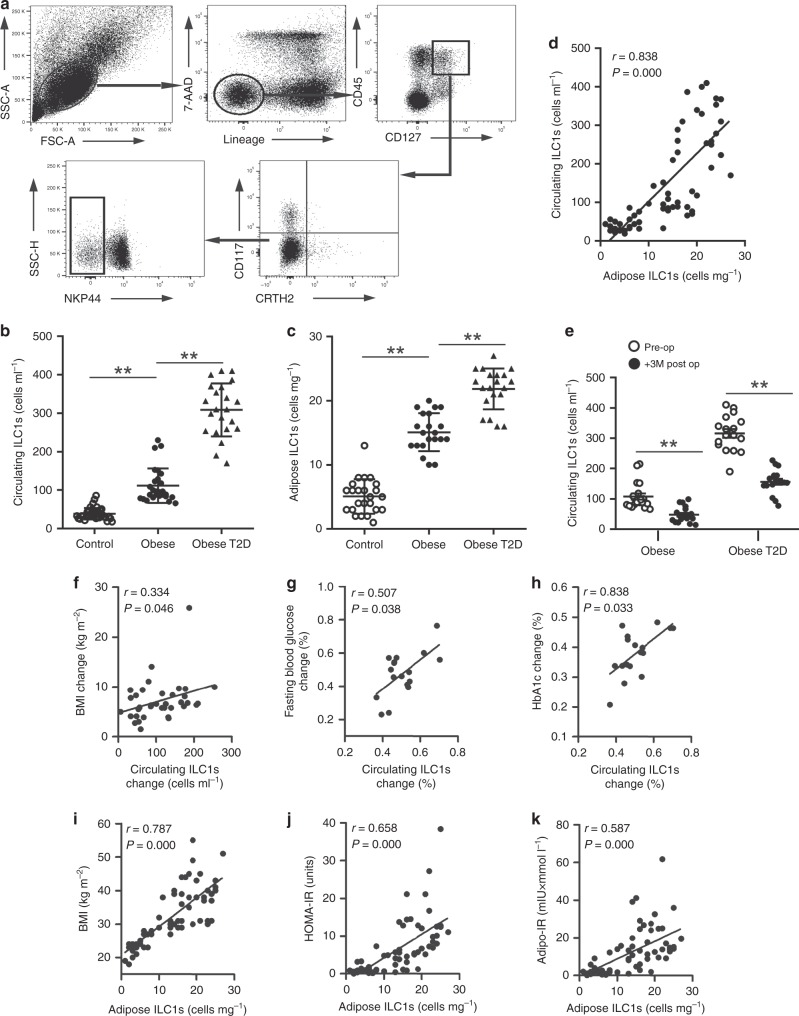
Adipose ILC1s correlate with the development of obesity and obese T2D. **a** Analysis of ILC1s by flow cytometry. **b** Absolute numbers (cells ml^−1^) of circulating ILC1s in control subjects (*n* = 36), obese subjects (*n* = 27), and obese T2D patients (*n* = 22). **c** Absolute numbers (cells mg^−1^) of adipose ILC1s in controls (*n* = 24), obese subjects (*n* = 21), and obese T2D patients (*n* = 20). **d** Correlation between adipose ILC1s numbers and circulating ILC1s (*n* = 65). **e** Numbers of circulating ILC1s before and at 3 months after bariatric surgery. **f** Correlation between reduction of circulating ILC1s and decrement of BMI levels in obese patients with 3-months follow-up (*n* = 36). **g**, **h** Correlations between decrement of fasting glucose levels and HbA1c levels with reduction of circulating ILC1s in obese T2D subgroup (*n* = 17). **i**–**k** Correlations between BMI, HOMA-IR, and Adipo-IR with adipose ILC1s (*n* = 65). ^**^P < 0.01 (two-way ANOVA tests followed by Bonferroni post hoc test). Error bars indicate s.d. ILC innate lymphoid cells

Circulating and adipose ILC1s were identified as Lin^−^CD45^+^ CD127^+^ CD117^−^CRTH2^−^NKP44^−^ lymphocytes (Fig. 1a), with isotype control data shown in Supplementary Fig. 1a. Compared with the controls, the absolute numbers of circulating ILC1s (cells ml^−1^) were significantly increased in obese subjects, which were further higher in obese T2D patients (Fig. 1b). Clinical characteristics of subjects with adipose tissue samples analyzed are summarized in Supplementary Table 1. Significantly higher numbers of ILC1s resident in the omental adipose tissue (cells mg^−1^) were detected in the obese group compared with the control group (15 ± 3 vs. 5 ± 3, *P* < 0.001, two-way ANOVA tests followed by Bonferroni post hoc test, Fig. 1c), which was even higher in obese T2D patients (obese T2D vs. obese: 23 ± 3 vs. 15 ± 3, *P* < 0.001, two-way ANOVA tests followed by Bonferroni post hoc test, Fig. 1c).

Meaningfully, a positive correlation between the number of circulating and adipose ILC1s was observed (r = 0.838, *P* = 0.000, Spearman’s correlation, *t* test, Fig. 1d). After controlling for the age and sex, circulating ILC1s were positively associated with fasting glucose levels (r = 0.713, *P* = 0.000, Spearman’s partial correlation, *t* test), postprandial blood glucose levels (r = 0.756, *P* = 0.000, Spearman’s partial correlation, *t* test), and HbA1c levels (r = 0.801, *P* = 0.000, Spearman’s partial correlation, *t* test). Furthermore, the numbers of adipose ILC1s were also positively related with fasting glucose levels (r = 0.677, *P* = 0.000, Spearman’s partial correlation, *t* test), postprandial blood glucose levels (r = 0.701, *P* = 0.000, Spearman’s partial correlation, *t* test), and HbA1c levels (r = 0.753, *P* = 0.000, Spearman’s partial correlation, *t* test), after adjusted for age and sex (Table 2). In 19 obese subjects and 17 obese T2D patients with 3 months of follow-up (Supplementary Table 2), compared with their baseline levels, the numbers of circulating ILC1s were all significantly reduced after 3 months post surgery (Fig. 1e). Importantly, in all obese subjects with 3 months of follow-up, the reduction of circulating ILC1s correlated with the decrement of body mass index (BMI) levels (r = 0.334, *P* = 0.046, Spearman’s correlation, *t* test, Fig. 1f). Moreover, in obese T2D subgroup, the reduction of circulating ILC1s positively correlated with decrement of fasting glucose levels (r = 0.507, *P* = 0.038, Spearman’s correlation, *t* test, Fig. 1g) and HbA1c levels (r = 0.838, *P* = 0.033, Spearman’s correlation, *t* test, Fig. 1h).

**Table 2:** Circulating and adipose ILC1s correlate with glycemic disturbance

	Circulating ILC1s (*N* = 85)		Adipose ILC1s (*N* = 65)	
	R	*P*	r	*P*
FBG (mmol l^−1^)	0.715	0.000	0.682	0.000
2 -h post (mmol l^−1^)	0.750^a^	0.000	0.706^b^	0.000
HbA1c (%)	0.790	0.000	0.755	0.000
*After adjusted for age and sex*				
FBG (mmol l^−1^)	0.713	0.000	0.677	0.000
2 -h post (mmol l^−1^)	0.756^a^	0.000	0.701^b^	0.000
HbA1c (%)	0.801	0.000	0.753	0.000

*FBG* fasting blood glucose, *2* *h post* 2 h postprandial blood glucose, *ILC* innate lymphoid cells

^a^*N* = 56

^b^*N* = 48

Relationship between adipose ILC1s and insulin resistance was further evaluated. As shown in Fig. 1i–k, adipose ILC1s positively correlated with BMI (r = 0.787, *P* = 0.000, Spearman’s correlation, *t* test), homeostasis model assessment of insulin-resistance (HOMA-IR) values (r = 0.658, *P* = 0.000, Spearman’s correlation, *t* test), and adipose tissue insulin-resistance index (Adipo-IR) (r = 0.587, *P* = 0.000, Spearman’s correlation, *t* test).

### Adipose ILC1s promote adipose fibrogenesis in humans

**Fig. 2:**
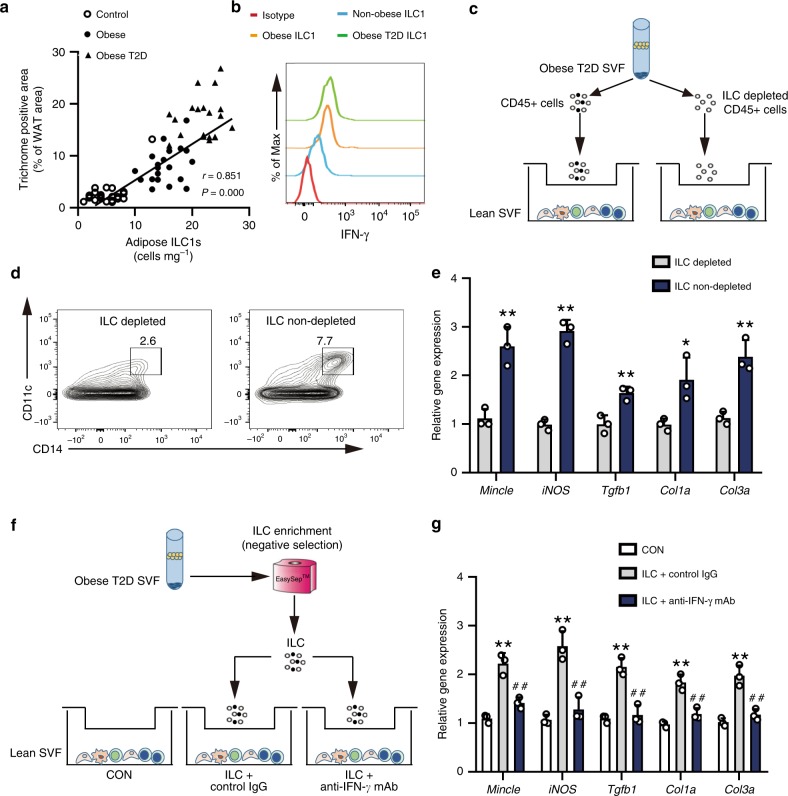
Adipose ILC1s contribute to adipose tissue fibrogenesis in humans. **a** Association of adipose ILC1s with trichrome C staining area. **b** Representative histogram of IFN-γ in adipose ILC1s from control subjects, obese subjects, and obese T2D patients. The data are representative of three independent experiments. **c**–**e** SVFs isolated from control subjects were co-cultured with either CD45^+^ cells or ILCs-depleted CD45^+^ cells sorted from the SVFs of obese T2D patients; meanwhile, trehalose-6,6’-dimycolate (5 μg well^−1^) and palmitate (200 μM) were added in the lower chamber. Three days after co-culture, SVFs in the lower chamber were collected for further detection. **c** Illustration of the co-culture experiments. **d** Representative plots of CD11c^+^ macrophages in SVFs of the lower chamber. **e** mRNA expression of *Mincle*, *iNOS*, and fibrosis-related genes (*Tgfb1*, *Col1a*, *Col3a*) in SVFs of the lower chamber. ^*^P < 0.05 vs. ILC depleted group; ^**^P < 0.01 vs. ILC depleted group (unpaired two-way Student’s *t* test). The data are representative of three independent experiments. **f**, **g** In another set of the co-culture experiment, 1 × 10^8^ cells of the SVF from obese T2D patients were magnetically enriched for ILCs using negative immunomagnetic selection. Then, adipose ILCs were co-cultured with SVFs of control subjects (1 × 10^6^ well^−1^). Trehalose-6,6’-dimycolate (5 μg well^−1^), palmitate (200 μM), recombinant human IL-12 (20 ng ml^−1^), and recombinant human IL-18 (20 ng ml^−1^) were supplemented in the upper chamber, with neutralizing IFN-γ antibody (20 ng ml^−1^) or IgG isotype control antibody (20 ng ml^−1^) added in separate group. SVFs isolated from adipose tissue of control subjects were cultured alone and determined as control group. After co-culture for 72 h, SVFs in the lower chamber were collected for further detection. **f** Graphical illustration of the co-culture experiments. **g** mRNA expression of *Mincle*, *iNOS*, *Tgfb1*, *Col1a*, and *Col3a* in SVFs of the lower chamber. ^**^P < 0.01 vs. control group; ^##^*P* < 0.01 vs. ILC+Control IgG group (two-way ANOVA tests followed by Bonferroni post hoc test). The data are representative of three independent experiments. Error bars indicate s.d. IFN interferon, ILC innate lymphoid cells

We next evaluated the potential role of adipose ILC1s in the development of adipose tissue fibrosis. Compared with the control subjects, obese individuals showed more collagen fibers around adipocytes in the adipose tissue (Supplementary Fig. 1b), which was further confirmed by a higher percentage of fibrotic area and higher expression levels of fibrotic-related genes (Supplementary Fig. 1c). The percentage of positively stained area indicated for adipose tissue fibrosis correlated with the number of adipose ILC1s (r = 0.851, *P* = 0.000, Spearman’s correlation, *t* test), BMI (r = 0.785, *P* = 0.000, Spearman’s correlation, *t* test), HOMA-IR (r = 0.714, *P* = 0.000, Spearman’s correlation, *t* test), and Adipo-IR (r = 0.658, *P* = 0.000, Spearman’s correlation, *t* test) (Fig. 2a; Supplementary Table 3). Multivariate stepwise regression analysis further revealed that the number of adipose ILC1s was the major determinant of the variations of adipose fibrosis level (β = 0.689, *P* = 0.000) (adjusted R^2^ for the model = 0.728) (Table 3).

**Table 3:** Multivariate stepwise regression analysis

	B	Standard error	Standard β	*P-*value
*Positive stained area*				
Adipose ILC1s	0.689	0.130	0.779	0.000
BMI	−0.087	0.103	−0.106	0.403
Adipo-IR	−0.152	−0.104	−0.221	0.149
HOMA-IR	0.555	0.269	0.373	0.044

*BMI* body mass index, *HOMA-IR* homeostasis model assessment-insulin resistance

Notably, compared with the controls, higher expressions of IFN-γ were found in adipose ILC1s from obese subjects, while adipose ILC1s from obese subjects and obese T2D patients expressed similar levels of IFN-γ (Fig. 2b). To address the role of adipose ILC1s in adipose tissue fibrogenesis, stromal vascular fractions (SVFs) from control subjects were co-cultured with either CD45^+^ cells or ILCs depleted CD45^+^ cells sorted from the SVFs of obese T2D patients (Fig. 2c; Supplementary Fig. 1d); meanwhile, both trehalose-6,6’-dimycolate, a mycobacterial cell wall glycolipid that is known to be a macrophage-inducible c-type lectin (Mincle) ligand^17^, and palmitate were added to mimic the in vivo microenvironment. Three days after co-culture, higher numbers of CD11c^+^ macrophages (Fig. 2d; Supplementary Fig. 1e) and significantly increased mRNA expression of *Mincle*, inducible nitric oxide synthase (*iNOS*), and transforming growth factor beta-1 (*Tgfb1*) were observed in SVFs of the ILCs non-depleted group relative to those in the ILCs depleted group (Fig. 2e). Moreover, mRNA expression of key collagen genes, *Col1a* and *Col3a*, was notably increased in SVFs of the ILCs non-depleted group (Fig. 2e).

To further confirm whether adipose ILC1s could promote adipose tissue fibrogenesis independent of other obesity-induced CD45^+^ cells, adipose ILCs were co-cultured with SVFs of control subjects (Fig. 2f). Three days after co-culture, expressions of fibrosis-related genes and ECM regulators, including *Mincle*, *iNOS*, *Tgfb1*, *Col1a*, and *Col3a*, were significantly increased in SVFs co-cultured with ILCs compared with those in the control group, while these changes were notably reversed by administration of neutralizing IFN-γ antibody (Fig. 2g). These findings indicate that the effect of adipose ILC1s on adipose tissue fibrogenesis is independent of other obesity-induced CD45^+^ cells and is potentially mediated by IFN-γ.

### Reconstruction of the adipose ILC1 population induces adipose fibrosis

**Fig. 3:**
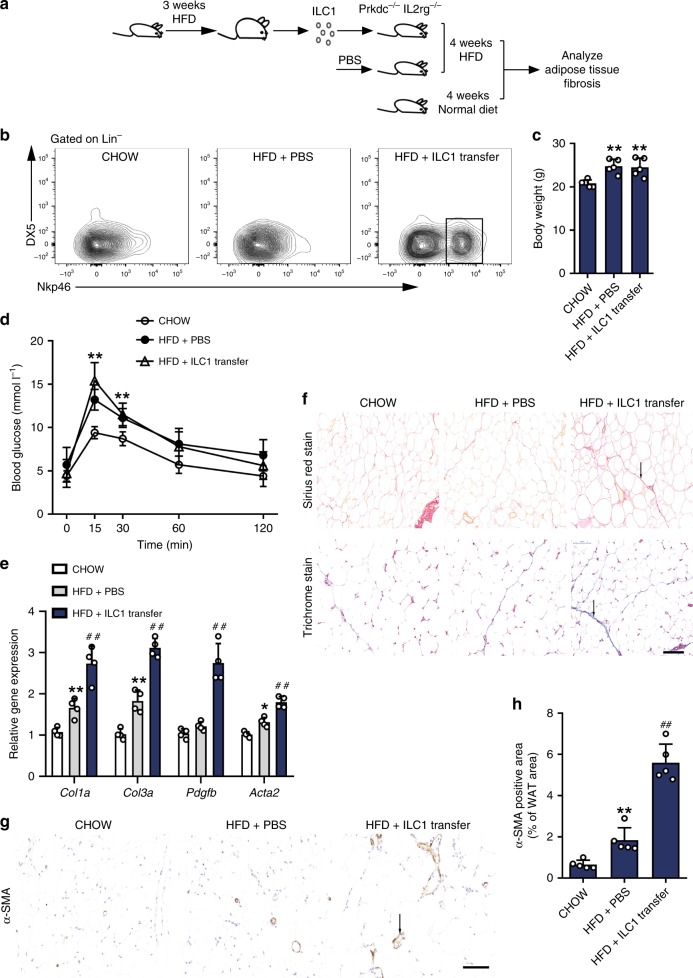
Adipose ILC1s contribute to obesity-associated adipose fibrosis. Briefly, C57BL/6 mice were fed a HFD for 3 weeks, and 5 × 10^4^ adipose ILC1s were sorted (>95% purity) from adipose tissue and adoptively transferred intravenously (i.v.) into *Prkdc*^*−/−*^*IL2rg*^*−/−*^ mice. The recipient mice were fed a HFD for 4 weeks. **a** Schematic of the experiment. **b** Representative plots indicate adoptively transferred adipose ILC1s in the VAT of recipient mice 4 weeks after transfer. **c** Body weight. **d** Glucose tolerance test. **e** Expression levels of fibrosis-related genes, assessed by q-PCR. **f** Representative Masson’s trichrome C and Sirius red staining in VAT of mice with different treatment (scale bar: 100 μm). **g**, **h** Representative αSMA staining (scale bar: 100 μm) and quantification of αSMA-positive area in VAT of mice with different treatment. Arrows indicate positive staining areas. ^*^P < 0.05 vs. CHOW group; ^**^P < 0.01 vs. CHOW group; ^##^*P* < 0.01 vs. HFD+PBS group (two-way ANOVA tests followed by Bonferroni post hoc test). The data are representative of three independent experiments, with *n* = 4–6 mice per group. Error bars indicate s.d.

To further determine the causative role of adipose ILC1s in adipose tissue fibrosis, we adoptively transferred purified adipose ILC1s from HFD-fed wild-type mice into *Prkdc*^*−/−*^*IL2rg*^*−/−*^ mice and fed HFD for 4 weeks (Fig. 3a). Adipose ILC1s in visceral adipose tissue (VAT) were identified as Lin^−^NK1.1^+^NKP46^+^T-bet^+^Eomes^−^DX5^−^ lymphocytes (Supplementary Fig. 1f), as reported recently^11^. Four weeks after cell transfer and HFD feeding, we observed that adipose ILC1s significantly accumulated in recipient VAT of mice receiving adipose ILC1 transfer compared with untreated control mice fed with normal diet (CHOW) or HFD (Fig. 3b). There was no appreciable difference in body weight between mice receiving ILC1 transfer and HFD mice receiving PBS injection (Fig. 3c), whereas glucose intolerance tended to be more severe in mice receiving ILC1 transfer (Fig. 3d). Expression of key collagen genes, *Col1a* and *Col3a*, was significantly higher in adipose tissue of mice receiving ILC1 transfer (Fig. 3e). In addition, expression of platelet derived growth factor subunit b (*Pdgfb*) and alpha-smooth muscle actin (*Acta2*), which regulates fibroblast proliferation and encodes the myofibroblast marker α-smooth muscle actin (*αSMA*), respectively, was significantly increased in mice receiving ILC1 transfer (Fig. 3e). Masson’s trichrome and Sirius red staining further revealed extensive interstitial fibrosis in VAT of mice receiving ILC1 transfer relative to HFD mice receiving PBS injection (Fig. 3f). In agreement, there was a significant increase in the area of αSMA-positive cells in mice receiving ILC1 transfer relative to HFD mice receiving PBS injection, as assessed by immunohistological analyses (Fig. 3g, h). In addition, the hepatic triglyceride content and serum-free fatty acid concentrations tended to increase in mice receiving ILC1 transfer than in HFD mice receiving PBS injection, which did not reach statistical significance (Supplementary Fig. 1g, h).

### Adipose ILC1s regulate ATMs and TGF-β1 signaling

**Fig. 4:**
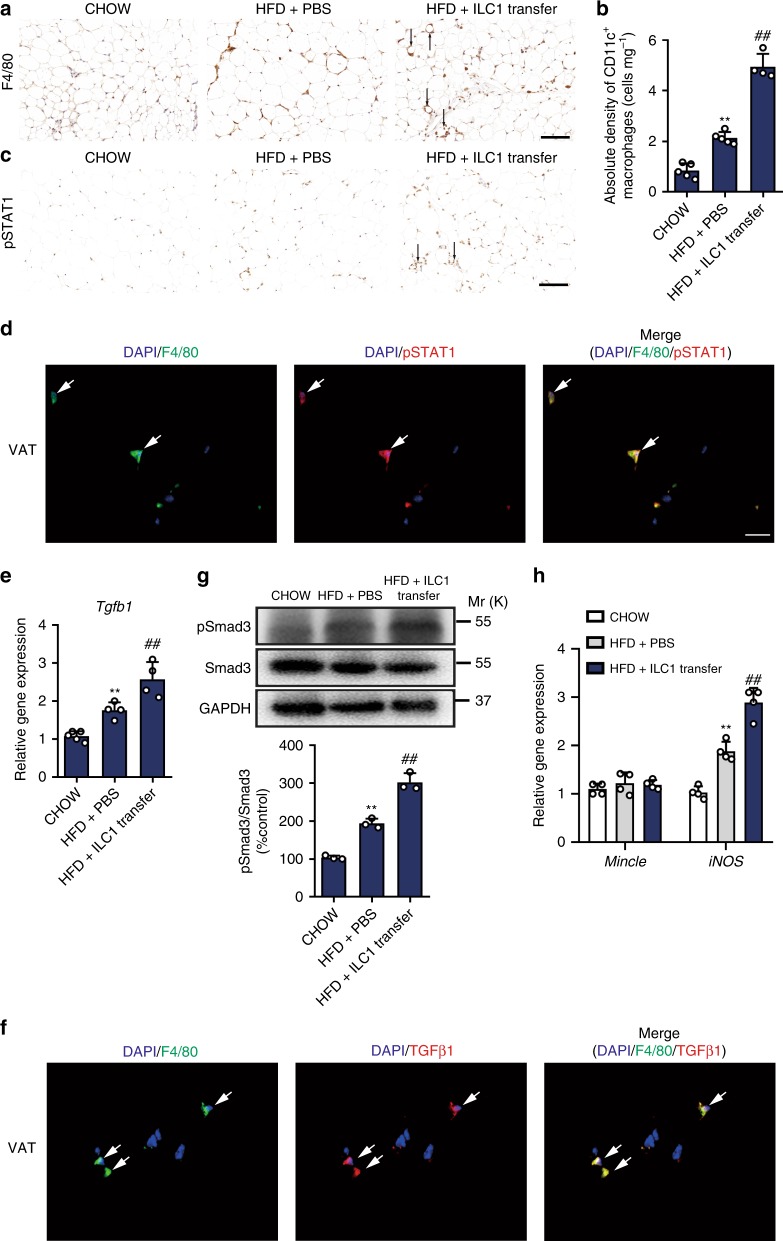
Adipose ILC1s regulate ATMs and activate TGF-β1 signaling pathway. C57BL/6 mice were fed a HFD for 3 weeks, and 5 × 10^4^ adipose ILC1s were sorted and adoptively transferred into *Prkdc*^*−/−*^*IL2rg*^*−/−*^ mice. The recipient mice were fed a HFD for 4 weeks. **a** Representative microscopic images with F4/80 staining in VAT of mice with different treatment (scale bar: 100 μm). **b** Absolute density (cells mg^−1^) of CD11c^+^ macrophages was analyzed in the VAT of recipient mice compared with CHOW or HFD controls. **c** Representative microscopic images with phosphorylated STAT1 staining in VAT of mice with different treatment (scale bar: 100 μm). **d** VAT specimens of mice receiving ILC1 transfer were costained for F4/80 (green), pSTAT1 (red), and DAPI (blue). Colocalization of F4/80 and pSTAT1 is shown in yellow in the merged image (scale bar: 20 μm). White arrows indicate typical stained cells. **e** Relative mRNA expression of *Tgfb1*. **f** VAT specimens of mice receiving ILC1 transfer were costained for F4/80 (green), TGF-β1 (red), and DAPI (blue). Colocalization of F4/80 and TGF-β1 is shown in yellow in the merged image (scale bar: 20 μm). White arrows indicate typical stained cells. **g** Phosphorylation and total levels of Smad3 in VAT of mice with different treatment. The phosphorylation levels of Smad3 were normalized to endogenous Smad3 levels, and are expressed as a percentage to those of CHOW group. **h** Relative mRNA expression of *Mincle* and *iNOS*. ^**^P < 0.01 vs. CHOW group; ^##^*P* < 0.01 vs. HFD+PBS group (two-way ANOVA tests followed by Bonferroni post hoc test). The data are representative of three independent experiments, with *n* = 4–6 mice per group. Error bars indicate s.d.

The appearance of crown-like structures (CLS) mainly composed of clusters of CD11c^+^ macrophages is associated with adipose tissue fibrosis^17^. Following HFD feeding, CLS was visualized in adipose tissue by IHC with staining for F4/80, while we found that mice receiving adipose ILC1 transfer and HFD feeding had substantially more CLS than did HFD mice receiving PBS injection (Fig. 4a). Accordingly, we detected higher numbers of CD11c^+^ and CD11c^+^CD206^+^ macrophages in mice receiving ILC1 transfer compared with HFD mice receiving PBS injection (Fig. 4b; Supplementary Fig. 2a, b), in line with increased phosphorylation levels of STAT1 (Fig. 4c), which is associated with proinflammatory CD11c^+^ macrophage activation^19^. Interestingly, immunofluorescence of pSTAT1 and the macrophage marker F4/80 showed pSTAT1 staining mostly colocalized with F4/80, which indicates that pSTAT1 is specifically expressed in ATMs (Fig. 4d). In vitro co-culture experiments using adipose ILC1s and bone marrow-derived macrophages (BMDMs) showed that, expressions of proinflammatory macrophage markers, *IL-6*, *NOS2*, and *TNF-α* were significantly increased in BMDMs co-cultured with isotype IgG treated adipose ILC1s compared with the control group, whereas these changes were notably reversed after administration of neutralizing IFN-γ antibody (Supplementary Fig. 2c).

Notably, TGF-β1 mRNA expression was markedly higher in VAT of mice receiving ILC1 transfer than that in HFD mice receiving PBS injection (Fig. 4e). Intriguingly, immunofluorescence showed that TGF-β1 staining was mostly colocalized with F4/80 (Fig. 4f). Moreover, TGF-β1 activity, as determined by phosphorylation of Smad3, was enhanced after ILC1 transfer (Fig. 4g). In agreement with elevated *iNOS* expression in human co-culture experiment, upregulation of *iNOS* expression was also observed in VAT of mice receiving ILC1 transfer; in contrast, *Mincle* mRNA expression tended to be higher in mice receiving ILC1 transfer, but did not reach significance (Fig. 4h).

### Adipose ILC1s induce adipose fibrosis via IFN-γ production

**Fig. 5:**
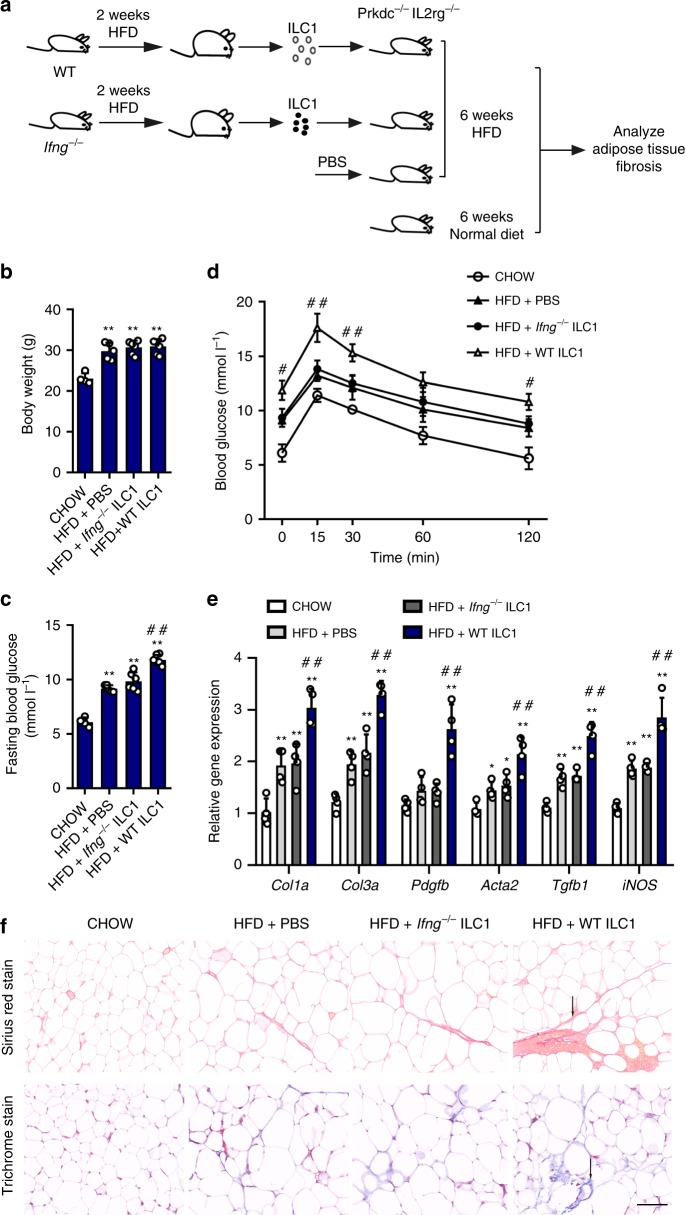
Adipose ILC1s induce adipose fibrosis through IFN-γ production. C57BL/6 and *Ifng*^*−/−*^ mice were fed a HFD for 2 weeks, and 5 × 10^4^ adipose ILC1s were sorted (>95% purity) from adipose tissue of each group and adoptively transferred i.v. into separate *Prkdc*^*−/−*^*IL2rg*^*−/−*^ mice. The recipient mice were fed a HFD for 6 weeks. Three weeks after HFD, adipose ILC1s were adoptively transferred into recipients again. **a** Schematic of the experiment. **b** Body weight. **c** Fasting blood glucose. **d** Glucose tolerance test. **e** Expression levels of fibrosis-related genes, assessed by q-PCR. **f** Representative Masson’s trichrome C and Sirius red staining in VAT of mice with different treatment (scale bar: 100 μm). Arrows indicate positive staining areas. ^*^P < 0.05 vs. CHOW group; ^**^P < 0.01 vs. CHOW group; ^#^*P* < 0.05 vs. HFD+*Ifng*^*−/−*^ ILC1 group; ^##^*P* < 0.01 vs. HFD+*Ifng*^*−/−*^ ILC1 group (two-way ANOVA tests followed by Bonferroni post hoc test). The data are representative of two independent experiments, with *n* = 4–6 mice per group. Error bars indicate s.d.

To validate that adipose ILC1s-derived IFN-γ was responsible for adipose tissue fibrogenesis and glycemic intolerance in obesity, we adoptively transferred adipose ILC1s from HFD-fed *Ifng*^*−/−*^ mice or wild-type mice into *Prkdc*^*−/−*^*IL2rg*^*−/−*^ mice and fed HFD for 6 weeks (Fig. 5a). Although no significant difference in body weight was found (Fig. 5b), mice receiving ILC1 transfer showed higher fasting glucose level and more severe glycemic intolerance compared with control mice fed with normal diet or HFD mice receiving PBS injection (Fig. 5c, d). However, following *Ifng*^*−/−*^ ILC1 transfer and HFD feeding in *Prkdc*^*−/−*^*IL2rg*^*−/−*^ mice, no significant difference in fasting glucose level or aggravating glycemic intolerance was found compared with HFD mice receiving PBS injection (Fig. 5c, d). Furthermore, expression of fibrosis-related genes and ECM regulators, including *Col1a*, *Col3a*, *Acta2*, *Tgfb1*, *Pdgfb*, and *iNOS* in VAT, was significantly increased in mice receiving ILC1 transfer compared to HFD mice receiving PBS injection and mice receiving *Ifng*^*−/−*^ ILC1 transfer (Fig. 5e). Masson’s trichrome and Sirius red staining further showed extensive interstitial fibrosis in VAT of mice receiving ILC1 transfer relative to HFD mice receiving PBS injection and mice receiving *Ifng*^*−/−*^ ILC1 transfer (Fig. 5f). These findings demonstrate that IFN-γ production mediates the effects of adipose ILC1s on adipose tissue fibrogenesis and glycemic disturbance.

### Inhibiting adipose ILC1 accumulation attenuates fibrosis

**Fig. 6:**
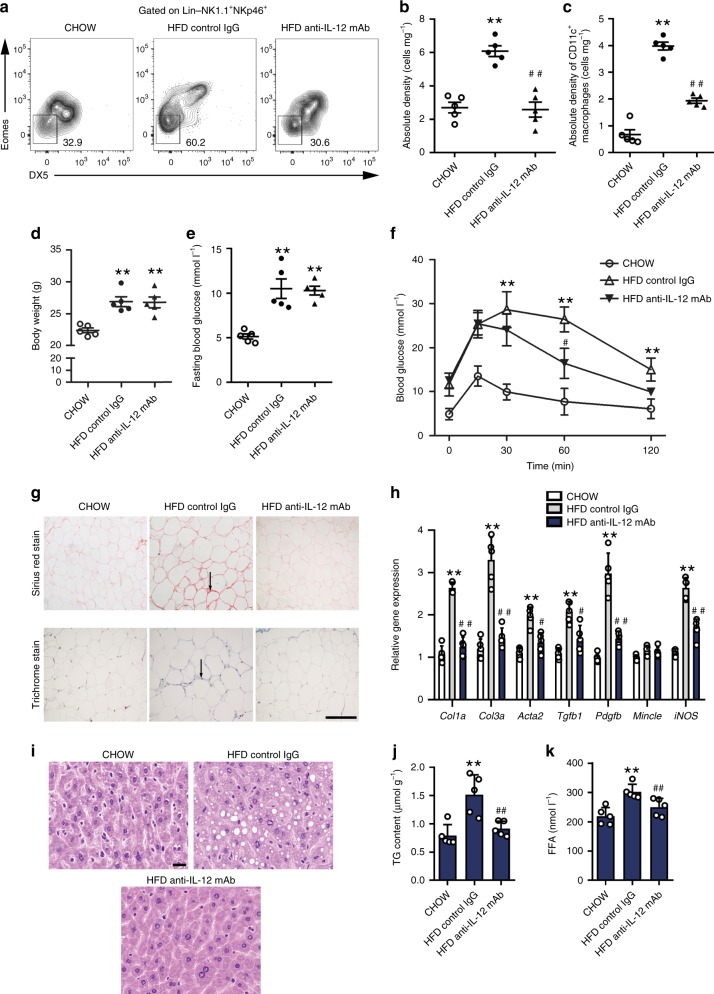
Inhibiting adipose ILC1s accumulation attenuates adipose fibrosis. *Rag1*^*−/−*^ mice were fed with normal diet or HFD, while mice in the HFD group were randomized into two groups of HFD anti-IL-12 mAb group and HFD control IgG group and continued with HFD feeding for another 4 weeks. HFD anti-IL-12 mAb group received rat anti-murine anti-IL-12p35 mAb administration via intraperitoneal injection at 250 μg per mouse every 3 days for 4 weeks; HFD control IgG group received the same dose of control IgG. **a** Representative plots indicate adipose ILC1s in the VAT of mice with different treatment. **b** Absolute density (cells mg^−1^) of adipose ILC1s in VAT. **c** Absolute density of CD11c^+^ macrophages (cells mg^−1^) in VAT. **d** Body weight. **e** Fasting blood glucose. **f** Glucose tolerance test. **g** Representative Masson’s trichrome C and Sirius red staining in VAT of mice with different treatment (scale bar: 100 μm). Arrows indicate positive staining areas. **h** Expression levels of fibrosis-related genes, assessed by q-PCR. **i** Representative microscopic images with H&E staining in the liver (scale bar: 20 μm). **j**, **k** Hepatic triglyceride levels and serum free fatty acid (FFA) levels. ^**^P < 0.01 vs. CHOW group; ^#^*P* < 0.05 vs. HFD+control IgG group; ^##^*P* < 0.01 vs. HFD+control IgG group (two-way ANOVA tests followed by Bonferroni post hoc test). The data are representative of two independent experiments, with *n* = 5 mice per group. Error bars indicate s.d.

IL-12 signaling is required for the proliferation of adipose ILC1s during diet-induced obesity^11^. To investigate the therapeutic potential of targeting adipose ILC1s to improve adipose tissue fibrosis and glycemic intolerance in obesity, IL-12 neutralizing antibody was utilized to block IL-12 signaling activation and subsequent adipose ILC1s accumulation in HFD-fed *Rag1*^*−/−*^ mice. Flow-cytometric analyses showed that adipose ILC1s in VAT were 2.5-fold higher after HFD feeding than in normal chow fed mice, whereas anti-IL-12 mAb treatment in HFD-fed mice significantly decreased numbers of adipose ILC1s (Fig. 6a, b). Consistently, CD11c^+^ and CD11c^+^CD206^+^ macrophages in VAT were also reduced after anti-IL-12 mAb administration; in contrast, CD206^+^ macrophages were unchanged in anti-IL-12 mAb-treated HFD-fed mice compared with control IgG treatment (Fig. 6c; Supplementary Fig. 2d). Although body weight and fasting blood glucose were not affected (Fig. 6d, e), improved glucose intolerance was observed in anti-IL-12 mAb-treated mice, compared with mice treated with control IgG (Fig. 6f). Strikingly, abnormal collagen deposition was notably reduced in VAT of anti-IL-12 mAb-treated mice, as shown by Masson’s and Sirius Red staining (Fig. 6g). Meanwhile, expression of fibrosis-related genes and ECM regulators, including *Col1a*, *Col3a*, *Acta2*, *Tgfb1*, and *Pdgfb*, was significantly decreased after anti-IL-12 mAb treatment (Fig. 6h). The beneficial effect of anti-IL-12 mAb treatment on interstitial fibrosis was also evidenced by reduced gene expression of *iNOS*, in accordance with decreased CD11c^+^ macrophages (Fig. 6h). In addition, histological examination revealed that hepatic steatosis was markedly attenuated in anti-IL-12 mAb-treated mice relative to mice treated with control IgG (Fig. 6i). Consistently, hepatic triglyceride content and serum-free fatty acid concentrations were significantly reduced in anti-IL-12 mAb-treated mice compared with mice treated with control IgG (Fig. 6j, k).

To further validate the potential of targeting adipose ILC1s to improve adipose tissue fibrogenesis and glycemic intolerance in obesity, IL-12 neutralizing antibody was administrated in HFD-fed wild-type mice. Compared with mice treated with control IgG, improved glucose intolerance was observed in anti-IL-12 mAb-treated wild-type mice (Supplementary Fig. 2e). Moreover, anti-IL-12 mAb treatment significantly decreased expression of fibrosis-related genes and ECM regulators including *Col1a*, *Col3a*, *Acta2*, *Tgfb1*, *Pdgfb*, and *iNOS* in VAT of anti-IL-12 mAb-treated wild-type mice (Supplementary Fig. 2f). In addition, Masson’s trichrome and Sirius red staining revealed abnormal collagen deposition in VAT of HFD-fed mice treated with control IgG, whereas anti-IL-12 mAb-treated wild-type mice showed significantly less fibrotic changes (Supplementary Fig. 2g).

## Discussion

Whether adipose ILCs contribute to the development of obese T2D in humans and the underlying mechanisms remain unclear. In this study, we characterize adipose ILC1s as a pathophysiology factor linking obesity and T2D. More importantly, through a comprehensive set of experiments in humans and mice, we demonstrated that adipose ILC1s promote adipose tissue fibrogenesis via increasing CD11c^+^ macrophages and activating the TGF-β1/Smad3 signaling pathway.

This study provides evidences that adipose ILC1s correlated with the development of obese T2D in humans. In this study, the absolute density (cells mg^−1^) of adipose ILC1s was investigated, since it has been reported that other adipose-resident cells, such as natural killer T (NKT) cells, ILC2, and regulatory T cells (Tregs) are reduced in the VAT of HFD-fed mice and affect the frequencies of minor cell populations^20^. A role of adipose ILC1s in the development of T2D was elucidated by the findings that adipose tissue of obese T2D patients contained significantly higher numbers of adipose ILC1s than that of obese and control subjects (Fig. 1), and further reinforced by correlations of adipose ILC1s with glycemic parameters either before or after adjustments for confounding factors (Table 2). Notably, a tight association was observed between adipose ILC1s and circulating ILC1s, with the reduction of circulating ILC1s positively correlated with the improvement of glycemic parameters in obese T2D patients after 3 months of bariatric surgery (Fig. 1). These data also indicate that increased levels of circulating ILC1s might act as a circulating marker of adipose dysfunction and T2D. These data expand our knowledge of adipose ILC1s and provide clinical evidences that adipose ILC1s contribute to the development of obese T2D in humans.

Another important finding of this study is the characterization of a previously unrecognized role of adipose ILC1s in adipose tissue fibrogenesis. Our data showed that adipose ILC1s positively correlated with extents of fibrosis in human omental adipose tissue and represented a major contributor to adipose tissue fibrogenesis as depicted in a multivariate regression model (Fig. 2, Table 3). Importantly, a direct role of adipose ILC1s in adipose tissue fibrogenesis was observed in our in vitro co-culture experiments, in which SVFs from control subjects were co-cultured with either CD45^+^ cells or ILCs depleted CD45^+^ cells sorted from the SVFs of obese T2D patients (Fig. 2). To exclude the role of CD8^+^ T cells and NK cells which have been reported to express IFN-γ in obese VAT^21,22^, adipose ILCs were magnetically enriched from obese T2D patients and co-cultured with SVFs of control subjects. Expressions of fibrosis-related genes and ECM regulators were significantly increased in SVFs after co-cultured with ILCs, indicating that adipose ILC1s can directly promote adipose tissue fibrogenesis and are independent of other obesity-induced CD45^+^ cells (Fig. 2). Of note, administration of neutralizing IFN-γ antibody markedly reversed the upregulation of genes related to fibrogenesis in SVFs co-cultured with adipose ILCs (Fig. 2), further confirming that IFN-γ production mediates the effect of adipose ILC1s on adipose tissue fibrogenesis. In addition, since IFN-γ expression from adipose ILC1s was similar between obese subjects and obese T2D patients (Fig. 2), co-culture experiments using ILCs from SVFs of obese subjects were expected to obtain similar results to obese T2D patients. Therefore, no further co-culture experiments were performed. In support of our findings in humans, adoptive transfer of adipose ILC1s into *Prkdc*^*−/−*^*Il2rg*^*−/−*^ mice aggravated adipose tissue fibrosis in the absence of complicated influences of T cells and NK cells, further demonstrating that adipose ILC1s play a causative role in the development of adipose tissue fibrosis (Fig. 3).

To validate that adipose ILC1s-derived IFN-γ was responsible for adipose tissue fibrogenesis and glycemic intolerance during HFD feeding, adipose ILC1s from HFD-fed *Ifng*^*−/−*^ mice or wild-type mice were adoptively transferred into recipient mice. Consistently, mice receiving ILC1 transfer showed higher fasting glucose level, more severe glycemic intolerance, and aggravated adipose fibrosis compared with HFD mice receiving PBS injection (Fig. 5). However, following *Ifng*^*−/−*^ ILC1 transfer into recipients, no significant differences were found in these glycemic and adipose fibrosis parameters compared with HFD mice receiving PBS injection (Fig. 5). In addition, we utilized antibody depletion methods to inhibit adipose ILC1s accumulation and observed a notably remission of adipose fibrosis, suggesting the potential of targeting adipose ILC1s to improve adipose tissue fibrosis and glycemic intolerance in obesity (Fig. 6). Importantly, these beneficial effects of IL-12 neutralizing antibody treatment were also observed in intact wild-type mice, not only in immunodeficient mice (Supplementary Fig. 2). Collectively, these results provided direct and solid evidences supporting the role of IFN-γ production by adipose ILC1s in promoting adipose tissue fibrogenesis and provide insights into the pathogenic mechanism of local immune disturbances in obesity-associated metabolic disturbance.

Recently, ATMs are reported to play a key role in regulating adipose tissue fibrogenesis^16–18^, promoting us to explore the role of adipose ILC1s in regulating ATMs function in humans. We found that CD11c^+^ macrophages were increased in SVFs co-cultured with CD45^+^ cells containing adipose ILC1s (Fig. 2), thus providing evidences that adipose ILC1s could regulate CD11c^+^ macrophage activation in humans. A recent study using IFN-γ reporter mice showed that IFN-γ is predominantly produced by ILC1s in adipose tissue, via which adipose ILC1s promote proinflammatory CD11c^+^ macrophage activation^11^. Consistent with these previous findings^17^, increased CD11c^+^ and CD11c^+^CD206^+^ macrophages, and CLS density were observed in VAT of mice receiving ILC1 transfer (Fig. 4). More importantly, we provided evidences that adipose ILC1 from obese subjects expressed high levels of IFN-γ (Fig. 2). The role of adipose ILC1-derived IFN-γ in macrophage activation was further reinforced by the in vitro co-culture experiments, in which adipose ILC1s were found to promote BMDMs activation toward proinflammatory phenotype, depending on IFN-γ production (Supplementary Fig. 2). TGF-β1/Smad3 activation contributes to persistent and aberrant ECM remodeling of VAT in obesity, and modulation of TGF-β1 activity might be an effective treatment strategy for obesity and related diabetes^23,24^. In line with previous studies that macrophage infiltrated areas are in rich of TGF-β1 expression in VAT of obese mice^23^, VAT of mice receiving ILC1 transfer showed increased mRNA expression of *Tgfb1* (Fig. 4). Accordingly, activated TGF-β1 pathway, as evidenced by increased phosphorylation of pSmad3, was also observed (Fig. 4). Importantly, our in vitro co-culture experiments also delineate that adipose ILC1 act to increase *Tgfb1* expression in SVFs (Fig. 2). These data thus demonstrate that induction of adipose tissue fibrosis by adipose ILC1 is related to the regulation of macrophage and activation of the TGF-β1/pSmad3 signaling pathway.

Fibrogenesis is a complex process caused by a variety of cells, such as myofibroblasts, macrophages, and parenchymal cells. Previous studies showed that myofibroblasts are derived from different cell types including resident fibroblasts and fibrocytes, depending on different tissues or organs^25^. In adipose tissue, interaction between *Mincle* expressing macrophages and fibroblast might promote myofibroblast formation^17^. In our in vitro co-culture experiments, expression of *Mincle* was increased after stimulation with trehalose-6,6’-dimycolate and palmitate in SVFs co-cultured with CD45^+^ immune cells containing adipose ILC1s and in SVFs co-cultured with adipose ILCs (Fig. 2). Furthermore, increased number of αSMA-positive cells or myofibroblasts was observed in mice receiving ILC1s transfer (Fig. 3). Besides myofibroblasts, preadipocytes are also reported to be source of the ECM, and macrophage can increase the profibrogenesis function through *iNOS* expression and subsequent production of NO^16,18^. Here, increased expression of *iNOS* in both SVFs co-cultured with adipose ILC1s and VAT of mice receiving ILC1s transfer were observed (Figs. 2, 4). Hence, these findings indicate that both preadipocytes and myofibroblasts participate in the process of adipose ILC1s-associated fibrogenesis. In addition to macrophages, *iNOS* can be expressed by a variety of immune cell populations after induction by cytokines or other stimuli, including neutrophils^26^, mature dendritic cells^27^, and Th17 cells^28^, which have been reported to be markedly increased in obese subjects^5^. In this study, since the gene expression of *iNOS* was detected using adipose tissue homogenates rather than sorted CD11c^+^ macrophages, the *iNOS* reduction not entirely in accordance with the decrease of CD11c^+^ macrophage might due to other cell types’ contributions to *iNOS* expression in anti-IL-12 mAb-treated mice.

A recent study showed that the frequency of a subset of CD56^low^CD3^−^CD16^−^CD127^low^ ILC1-like cells was decreased in adipose tissue of extremely obese subjects^29^. Based on the limited clinical baseline data provided by that study, different extent of obesity (mean BMI: 48 vs. 37) and glycemic status (non T2D vs. T2D) of obese subjects might account for the discrepancy on human adipose ILC1s between that study and our study, apart from the different markers in identification of adipose ILC1s. To reach a consensus on ILC1 identification and better clarify its role in human metabolic disease, follow-up studies including a larger clinical cohort with different stage of obesity and a strictly matched control cohort are required to evaluate the phenotypic and functional characteristics of adipose ILC1s.

In conclusion, our data clarify a direct role of adipose ILC1s in adipose tissue fibrogenesis and provide clues for understanding the pathogenic mechanism of local immune disturbances in obesity-associated metabolic disturbance. More importantly, our findings reveal that adipose ILC1s contribute to the development of obese T2D, thus providing a pathophysiological link between obesity and T2D. Adipose ILC1s may thus serve as a therapeutic target for the treatment or prevention of obese T2D.

## Methods

### Patients

A total of 85 individuals, including 49 obese patients underwent laparoscopic Roux-en-Y gastric bypass (RYGB) surgery, and 36 age- and sex-matched non-obese non-T2D controls received elective abdominal surgery (e.g., hernia or hemangioma resection) were enrolled from April 2017 to December 2017 at Drum Tower Hospital Affiliated to Nanjing University Medical School. All the enrolled 49 obese subjects had no history of diabetes and they received oral glucose tolerance test before surgery, in which 22 individuals were diagnosed as T2D according to American Diabetes Association criteria^30^. All participants completed questionnaires for medical history assessment and underwent anthropometric measurements. Patient height was measured by stadiometer with shoes off, to the nearest centimeter. The weight was measured on a digital scale with identical light clothing on, to the nearest 0.1 kg. The BMI was calculated as weight divided by height squared. Exclusion criteria were: acute infection in the past 3 months; stroke or myocardial infarction; autoimmune diseases, e.g., rheumatoid arthritis and systemic lupus erythematosus; malignant tumors; chronic digestive diseases, e.g., Crohn’s disease and colitis gravis; abnormal liver or renal dysfunction, with alanine transaminase (ALT) or aspartate transaminase (AST) 2.5-folds higher than the normal range, or estimated glomerular filtration rate (eGFR) <60 ml min^−1^ 1.73 m^−2^, based on the CKD-EPI equation; use of nonsteroidal or steroidal anti-inflammatory medicines in past 3 months; pregnancy; other endocrine system diseases, e.g., thyroid diseases and Cushing’s syndrome. Obese patients who underwent bariatric surgery were followed-up, and blood samples were collected from 19 obese patients and 17 obese T2D patients 3 months after surgery at the end of the study.

This study, including the use of human tissues was approved by the Ethics Review Committee of Nanjing Drum Tower Hospital Affiliated to Nanjing University Medical School (Approval number: 2017-030-02). All patients and control individuals meeting the inclusion criteria gave consent at the time of enrolment.

### Animal studies

All animal experimental protocols were approved by the Research Animal Care Committee of Drum Tower Hospital Affiliated to Nanjing University Medical School (Approval number: 2019AE01002), Nanjing, China. The following strains were used in this study: C57BL/6, *Rag1*^*−/−*^, *Ifng*^*−/−*^ (B6.129S7-Ifngtm1Ts/J, stock number 002287, purchased from the Jackson Laboratory), and *Prkdc*^*−/−*^*IL2rg*^*−/−*^ (NOD background, lacking mature T, B, and natural killer lymphocytes) mice. Experiments were conducted using age- and gender-matched mice in accordance with approved institutional protocols.

For antibody treatment experiments, 6-week-old male *Rag1*^*−/−*^ and C57BL/6 mice were obtained from Nanjing Biomedical Research Institute of Nanjing University and housed in a controlled environment (12 -h daylight cycle, lights off at 18:00) with food and water ad libitum. After 1 week of acclimation on a normal diet, mice were randomly divided into two groups and fed either a normal diet or a HFD (D12492, Research Diets). After 1 week, mice in the HFD group were randomized into two groups of HFD anti-IL-12 mAb group and HFD control IgG group, and continued with HFD feeding for another 4 weeks. HFD anti-IL-12 mAb group received rat anti-murine anti-IL-12p35 mAb (eBioscience, C18.2) administration via intraperitoneal injection at 250 μg per mouse every 3 days for 4 weeks; HFD control IgG group received the same dose of control IgG.

In adoptive transfer experiments, adipose ILC1s were purified from adipose tissue from C57BL/6 or *Ifng*^*−/−*^ mice fed a HFD by a flow cytometer (FACSAriaIII, BD, Bioscience) and transferred into *Prkdc*^*−/−*^*IL2rg*^*−/−*^ mice (6-week--old, purchased from Biocytogen Co., Ltd, Beijing, China) on HFD. After HFD feeding, recipient peripheral organs were harvested, and adoptively transferred cells were analyzed by flow cytometry.

All mice were weighed at the beginning to the feeding period and weekly thereafter until killed. Glucose tolerance test (GTT) was performed with an intraperitoneal injection of 10% D-glucose solution (2 g Kg^−1^ body weight) into the mice. Blood glucose levels were measured at 0, 15, 30, 60, and 120 min after glucose administration. Two days after GTT, all animals were anesthetized in chambers saturated with isoflurane and killed. Blood samples, VAT, and liver tissues were collected.

### Blood sample analysis

Fasting blood samples were also collected for HbA1c, triglycerides, total cholesterol, high-density lipoprotein cholesterol (HDL-C), low-density lipoprotein cholesterol (LDL-C), and free fatty acid (FFA) measurements. Plasma glucose amounts were assessed by the hexokinase method on TBA-200FR (Tokyo, Japan). Insulin concentration was evaluated by a chemiluminescence immunoassay (Cobas e601, Roche). HbA1c was quantitated by high-performance liquid chromatography (Bio-Rad D-10). Triglyceride, total cholesterol, HDL-C, and LDL-C concentrations were assessed by specific immunoassays (Cobas e601, Roche). Serum FFAs were detected via an enzymatic method (DiaSys Diagnostic Systems, Co., Ltd). HOMA-IR was derived as follows: [fasting serum insulin (mU/l) × fasting glucose (mmol/l)]/22.5^31^. The Adipo-IR was defined as fasting insulin concentration (mIU/ml) × fasting FFA (mmol/l)^32,33^.

Mouse serum was analyzed for FFA using a standard enzymatic assay following the manufacturer’s protocols (ColorfulGene biological technology Co., Ltd, Wuhan, China).

### Measurement of triglyceride content in the liver

For mouse liver triglyceride analysis, triglyceride content in the harvested liver tissue was measured using an enzymatic assay kit following the manufacturer’s protocols (JYM0190Mo, ColorfulGene biological technology Co., Ltd, Wuhan, China).

### Peripheral blood mononuclear cell isolation

PBMCs were isolated with vacutainer cell preparation tubes (Becton Dickinson, Franklin Lakes, NJ) from fasting blood samples in the non-obese and obese patients pre-surgery and in the obese patients after 3 months follow-up of surgery. Briefly, after centrifugation at 1800 × g for 30 min, cells in the resulting supernatants were collected, washed with phosphate-buffered saline (PBS), and frozen gradually within 8 h in freezing buffer containing 10% DMSO and 90% fetal bovine serum (FBS). Samples were stored in liquid nitrogen until further analysis.

### Adipose tissue biopsy and SVF preparation

Periumbilical adipose tissue samples at the omental region were obtained from 21 obese individials, 20 obese T2D patients, and 24 non-obese non-T2D control individuals perioperatively. Adipose tissue samples (~10 g) were collected and transported to laboratory immediately. For human SVFs preparation, fresh adipose samples were cut into small pieces and digested with 0.1% type II collagenase (Sigma-Aldrich, USA). After incubation for 1 h with shaking on a MACSmix Tube Rotator, the specimens were filtered through a 100-μm nylon mesh and centrifuged at 500×*g* for 10 min. The resulting supernatants were aspirated, and the pellets were resuspended in red blood cell (RBC) lysis buffer (GE, USA).

Mouse SVFs were prepared using 0.1% type I collagenase (Sigma-Aldrich, USA)^23^. In brief, VAT was physically dissociated using scissors and incubated for 1 h in digest solution (1 mg/ml type I collagenase in RPMI supplemented with 5% fetal calf serum, 1% L-glutamine, 1% penicillin–streptomycin, and 10 mM HEPES). Resulting dissociated tissue was passed through 100-μm nylon mesh, centrifuged, and adipocytes were removed from the supernatant. Red blood cells were lysed using RBC lysis buffer.

### Flow cytometry

Human PBMCs and SVFs were stained with the following antibodies for flow-cytometry analysis: CD3 (UCHT1, BD Biosciences, 1:200), CD19 (HIB19, BD Biosciences, 1:200), CD16 (B73.1, BD Biosciences, 1:100), CD45 (2D1, BD Biosciences, 1:100), CD127 (HIL-7R-M21, BD Biosciences, 1:50), CD117 (YB5, B8, BD Biosciences, 1:50), CRTH2 (BM16, BD Biosciences, 1:50), NKP44 (p44-8, BD Biosciences, 1:50), CD206 (19.2, BD Biosciences, 1:50), CD14 (M5E2, BD Biosciences, 1:100), IFN-γ (4S.B3, BD Biosciences, 1:50), CD11c (Bu15, BioLegend, 1:50), CD5 (L17F12, BioLegend, 1:100), TCRαβ (IP26, BioLegend, 1:100), and FCERIA (AER-37, BioLegend, 1:100). Human ILC1s were identified as a lineage negative for CD3, CD19, CD16, CD11c, CD5, TCRαβ and FCERIA (Lin^−^), CD45^+^, CD127^+^, CD117^−^, NKP44^−^, CRTH2^−^. Human macrophages were identified with antibodies against CD45, CD14, CD11c, and CD206 ^34^. 7-aminoactinomycin D (BD Biosciences) was used to gate live cells. For IFN-γ detection, intracellular cytokine staining was performed with the Cytofix/Cytoperm Plus kit (BD Biosciences). For animal studies, cell surface staining was performed using the following fluorophore-conjugated antibodies: TCRβ (H57-597, BD Biosciences, 1:100), CD3 (17A2, BD Biosciences, 1:200), CD19 (1D3, BD Biosciences, 1:200), TCRγδ (GL3, BD Biosciences, 1:100), Ly-6G (1A8, BD Biosciences, 1:100), F4/80 (BM8, BioLegend, 1:100), NK1.1 (PK136, BD Biosciences, 1:50), NKP46 (29A1.4, BD Biosciences, 1:50), DX5 (DX5, BD Biosciences, 1:50), Eomes (1219A, R&D Systems, 1:50), T-bet (4B10, BD Biosciences, 1:50), CD11b (M1/70, BD Biosciences, 1:100), CD11c (N418, BD Biosciences, 1:100), CD206 (C068C2, BioLegend, 1:100), CD45 (A20, BD Biosciences, 1:200). For analysis of cell surface molecules, cells were washed with PBS and resuspended in FACS buffer (2% FBS, 2 mM EDTA, 0.05% NaN_3_ in PBS), following by staining with the antibodies indicated in figures in FACS buffer for 30 min at 4 °C. After washing, the stained cells were analyzed on an LSR II Fortessa flow cytometer (BD, Bioscience) or sorted on a FACSAriaIII flow cytometer (BD Biosciences); the data were analyzed with FACSDiva software (BD, Bioscience) or Flowjo software version 9.6.4 (Tree Star, Inc.). The gating strategy for flow-cytometry analysis is shown in Supplementary Fig. 3.

### Co-culture experiments

As for the co-culture experiment, a transwell system was used (0.4-μm pore size semi-permeable membrane, BD Biosciences). In brief, CD45^+^ cells or Lin^−^CD127^+^-depleted CD45^+^ cells (5 × 10^5^ well^-1^) were sorted from SVFs isolated from obese T2D subjects, cultured in the upper chamber of the transwell insert in a 12-well plate, and SVFs isolated from adipose tissue of control subjects (1 × 10^6^ well^−1^) were seeded in the lower chamber of the transwell insert. Meanwhile, trehalose-6,6’-dimycolate (5 μg well^−1^, Nacalai Tesque, Kyoto, Japan) and palmitate (200 μM, Sigma) were added in the lower chamber. After co-culture for 72 h, SVFs in the lower chamber were collected for further detection.

In another set of co-culture experiment, 2 × 10^8^ cells of the SVF from obese T2D patients were magnetically enriched for ILCs using a negative selection kit that depletes non-ILCs, according to the manufacturer’s protocol (EasySep^TM^ Human ILCs Enrichment kit, STEMCELL Technologies). Then, adipose ILCs were cultured in the upper chamber of the transwell insert in a 12-well plate, and SVFs isolated from adipose tissue of control subjects (1 × 10^6^ well^−1^) were seeded in the lower chamber of the transwell insert. Meanwhile, trehalose-6,6’-dimycolate, palmitate, recombinant human IL-12 (R&D Systems), and recombinant human IL-18 (R&D Systems) were supplemented in the upper chamber, with neutralizing IFN-γ antibody (BioXCell) or IgG isotype control antibody (BioXCell) added in separate group. SVFs isolated from adipose tissue of control subjects were cultured alone and determined as control group. After co-culture for 72 h, SVFs in the lower chamber were collected for further detection.

As for the co-culture experiment using BMDMs and adipose ILC1s from C57BL/6 mice, detailed information were described on the Supplementary Methods section.

### RNA extraction and quantitative PCR

Adipose tissues, which were snap-frozen in nitrogen gas and stored at −80℃, and SVFs were homogenized in TRIzol Reagent (15596026, Life Technologies, Thermo Fisher Scientific) and mixed with chloroform. After spinning down, the upper aqueous phase was mixed with the same volume of 70% EtOH and applied to the RNeasy Mini Kit column according to the manufacturer’s instructions for the RNeasy Mini Kit (74104, QIAGEN). Extracted RNA was converted to cDNA (RR036A, TAKARA) according to the manufacturer’s instructions. Relative gene expression levels were analyzed by quantitative PCR on a Real-Time PCR system (LightCycler 480 II Roche, Switzerland). Fold differences in gene expression were calculated as 2^−△△Ct^ using β-actin as the housekeeping gene. Primers used are listed in Supplementary Table 4.

### Western blotting

For western blotting assays, adipose tissues were homogenized in lysis buffer (1% Triton X-100, 50 nM Tris HCl, 150 mM NaCl, 5 mM EDTA, 1 mM PMSF) with protease cocktail (Roche) and phosphatase inhibitors (1 mM Na_3_Vo_4_, 10 mM NaF). Equal amounts of protein (30 μg) were separated by gel electrophoresis, transferred onto a PVDF membrane (Millipore, Billerica, MA), and incubated with primary antibodies against phosphorylated Smad3 (ab52903, Abcam, 1:1000), and total Smad3 (ab40854, Abcam, 1:2000). GAPDH (ab8245, Abcam, 1:4000) was used as a loading control. The protein in the membrane was visualized using a Bio-Rad Clarity Western ECL Substrate (1705060, Bio-Rad). Uncropped western blot images can be found in the Source Data file.

### Immunohistological analysis

Adipose tissue samples (~200–300 mg) and the liver were washed with PBS, fixed in paraformaldehyde for 24 h, paraffin embedded, and sliced into 4-μm sections. The sections of adipose tissue were stained with Masson’s trichrome dye or 0.1% Sirius Red in saturated picric acid for fibrosis assessment. The presence of F4/80-positive macrophages, pSTAT1, and αSMA-positive area in VAT were detected. Immunohistochemistry was performed using the following antibodies: pSTAT1 (ab109461, Abcam, 1:50), F4/80 (ab6640, Abcam, 1:50), αSMA (ab7817, Abcam, 1:50). The sections of the liver were stained with hematoxylin and eosin. The quantitative histological analysis was performed by two investigators who had no knowledge of the origin of the slides. Staining images were captured and digitalized using an Olympus microscope (Olympus, Tokyo, Japan). Positive staining areas containing fibrillary collagens, as shown either with blue in Masson’s trichrome stain or red in Sirius Red stain, were quantified by NIH Image J software.

### Immunofluorescence

After deparaffinization and rehydration, adjacent 4-μm adipose tissue sections from mice were incubated with 10 mM citric acid and heated in a microwave to recover antigenicity. Permeabilization was performed in PBS with Triton X-100 and nonspecific binding was blocked with 10% normal goat serum in PBS. Adipose tissue sections were incubated with F4/80 antibody (ab6640, Abcam, 1:50), TGF-β1 antibody (ab92486, Abcam, 1:50), and pSTAT1 antibody (ab109461, Abcam, 1:50) in PBS with 3% BSA and 0.1% Tween-20 at 4 ℃ overnight. Tissue sections were incubated for 1 h at room temperature with Alexa Fluor 488-conjugated AffinPure goat anti-rabbit IgG (GB25303, Servicebio, 1:50) and Cy3-conjugated goat anti-rabbit IgG (GB21303, Servicebio, 1:50). The cell nuclei were visualized by the mounting solution with DAPI (G1012, Servicebio). Staining images were captured and digitalized using a Nikon Eclipse 80i microscope.

### Statistical analyses

All statistical analyses were performed using SPSS version 22.0 (SPSS Inc., USA). The data were presented as mean ± SD or *n* (%). Differences in continuous variables in two groups were determined by the Student’s *t* test, and differences in categorical variables were determined by the χ^2^ analysis or Fisher exact test. Group differences were compared with ANOVA tests for normally distributed variables, whereas nonparametric Mann–Whitney *U* test was performed for skewed parameters. Spearman’s bivariate correlation tests and partial correlation analyses were conducted to study the associations. *P-*values <0.05 were considered statistically significant.

### Reporting summary

Further information on research design is available in the Nature Research Reporting Summary linked to this article.

## Supplementary information

Supplementary information

Reporting Summary

Source Data

## Data Availability

Raw data for Figs. 1–6 and Supplementary Figs. 1 and 2 are presented in the Source Data file. All other data that support the findings of this study are available from the corresponding author upon reasonable request.
