# Supplementary material for: Adipose group 1 innate lymphoid cells promote adipose tissue fibrosis and diabetes in obesity
Source: Nat Commun. 2019 Jul 22;10:3254. doi: 10.1038/s41467-019-11270-1 (PMC6646407; doi:10.1038/s41467-019-11270-1)
Supplement: Supplementary file 1 — Supplementary information [file 41467_2019_11270_MOESM1_ESM.pdf]

Supplementary information

Wang et al.

Adipose group 1 innate lymphoid cells promote adipose tissue fibrosis and diabetes in obesity

Supplementary Tables

Supplementary Table 1 Characteristics of participants whose adipose tissue samples were analyzed

	Control	Obese	Obese T2D	*P* values			
				Overall	Control *vs.* Obese	Control *vs.* Obese T2D	Obese *vs.* Obese T2D
N	24	21	20	-	-	-	-
Age (years)	42.4±6.7	41.5±13.5	38.9±11.6	0.554	-	-	-
Male, n (%)	6(25.0)	5(23.8)	6(30.0)	0.896	-	-	-
BMI (kg m^{-2})	24.2±2.7	35.5±7.4	38.6±5.3	0.000	0.000	0.000	0.073
HbA1c (%)	5.3±0.5	5.7±0.3	8.8±1.2	0.000	0.076	0.000	0.000
Fasting glucose (mmol l^{-1})	4.7±0.4	5.3±0.5	11.0±3.5	0.000	0.304	0.000	0.000
2h blood glucose (mmol l^{-1})	5.7±0.9[a]	7.1±1.6	16.8±4.1	0.000	0.314	0.000	0.000
Fasting insulin (mIU ml^{-1})	5.7±7.6	21.6±13.7	28.7±14.2	0.000	0.000	0.000	0.066
HOMA-IR (units)	1.2±1.5	5.3±3.7	13.8±8.1	0.000	0.008	0.000	0.000
Triglycerides (mmol l^{-1})	1.2±0.5	1.8±0.8	4.3±3.6	0.000	0.394	0.000	0.000
Total cholesterol (mmol l^{-1})	4.4±0.7	4.7±0.8	5.1±1.1	0.038	0.390	0.012	0.095
HDL-C (mmol l^{-1})	1.2±0.3	1.1±0.3	1.0±0.1	0.007	0.170	0.002	0.068
LDL-C (mmol l^{-1})	2.6±0.6	2.9±0.6	2.6±0.6	0.208	-	-	-
Fasting FFA (mmol l^{-1})	0.45±0.1	0.7±0.1	0.7±0.1	0.000	0.000	0.000	0.130
Adipo-IR (mIU ml^{-1}×mmol l^{-1})	2.4±2.9	14.7±10.6	21.0±12.6	0.000	0.000	0.000	0.039

BMI, body mass index; HOMA-IR, homeostasis model assessment-insulin resistance; HDL-C, high-density lipoprotein cholesterol; LDL-C, low-density lipoprotein cholesterol; Adipo-IR, adipose insulin resistance index. All data are presented as mean±SD or n (%). Comparisons are by ANOVA and, when appropriate, LSD post-hoc test. [a], 13 non-obese control subjects were missing.

Supplementary Table 2 Clinical parameters of subjects pre-surgery and 3 months post-surgery

	Obese				Obese-T2DM				
	pre-surgery	3 months post-surgery	*P* value	Change	pre-surgery	3 months post-surgery	*P* value	Change	*P* for change
N	19	-	-	-	17	-	-	-	-
Age (years)	35.2±9.9	-	-	-	36.9±10.9	-	-	-	-
Male, n (%)	6(31.6)	-	-	-	3(17.6)	-	-	-	-
BMI (kg m^{-2})	38.4±6.9	31.8±5.2	0.002	6.6±7.3	38.7±4.9	31.8±4.0	<0.001	6.9±1.8	0.861
HbA1c (%)	5.7±0.9	5.1±0.3	0.023	0.5±1.0	8.9±1.4	5.5±0.7	<0.001	3.5±1.1	0.000
Fasting glucose (mmol l^{-1})	5.6±0.7	5.0±0.4	0.004	0.6±0.7	11.4±3.8	5.6±0.7	<0.001	5.8±3.6	0.000
2 h post (mmol l^{-1})	7.0±1.7	NA	-	-	17.1±4.5	6.8±1.9	<0.001	10.3±4.2	-
Fasting insulin (mIU ml^{-1})	26.4±11.0	11.6±3.7	<0.001	14.7±10.2	34.7±21.4	15.0±7.2	<0.001	19.7±16.0	0.265
HOMA-IR (units)	6.8±3.3	2.6±0.9	<0.001	4.2±3.1	16.6±9.5	3.6±1.5	<0.001	13.0±8.8	0.000
Triglycerides (mmol l^{-1})	1.8±0.7	1.1±0.3	<0.001	0.8±0.6	4.8±3.8	1.4±0.5	0.001	3.4±3.7	0.004
Total cholesterol (mmol l^{-1})	4.7±0.8	4.1±0.7	0.000	0.6±0.6	5.1±1.2	3.9±0.7	<0.001	1.3±0.9	0.012
HDL-C (mmol l^{-1})	1.2±0.3	1.2±1.4	0.229	0.1±0.2	1.0±0.1	1.0±0.2	0.498	0.0±0.2	0.675
LDL-C (mmol l^{-1})	2.9±0.7	2.2±0.6	0.000	0.7±0.7	2.9±0.6	2.2±0.6	0.125	0.3±0.7	0.136
Fasting FFA (mmol l^{-1})	0.6±0.1	0.4±0.2	0.000	0.2±0.2	0.7±0.1	0.4±0.1	<0.001	0.4±0.2	0.076
Adipo-IR (mIU ml^{-1}×mmol l^{-1})	17.0±9.1	4.3±2.6	<0.001	12.7±9.2	25.1±15.6	5.2±3.1	<0.001	19.9±14.1	0.075

BMI, body mass index; HOMA-IR, homeostasis model assessment-insulin resistance; HDL-C, high-density lipoprotein cholesterol; LDL-C, low-density lipoprotein cholesterol; Adipo-IR, adipose insulin resistance index. All data are presented as mean±SD or n (%). Comparisons are by ANOVA and, when appropriate, LSD post-hoc test. NA, not applicable.

Supplementary Table 3 Correlations between the percentage of positively stained area indicated for adipose tissue fibrosis and BMI, HOMA-IR, and Adipo-IR

	Trichrome positive area (% of WAT area)	
	r	*P*
BMI (kg m^{-2})	0.785	0.000
HOMA-IR (units)	0.714	0.000
Adipo-IR (mIU ml^{-1}×mmol l^{-1})	0.658	0.000

Supplementary Table 4 Primers used in this study

For human studies:

Number	Gene	Forward (5'-3')	Reverse (5'-3')
1	*COL1A1*	GAGGGCCAAGACGAAGACATC	CAGATCACGTCATCGCACAAC
2	*COL3A1*	GGAGCTGGCTACTTCTCGC	GGGAACATCCTCCTTCAACAG
3	*MMP-2*	TACAGGATCATTGGCTACACACC	GGTCACATCGCTCCAGACT
4	*MMP-9*	TGTACCGCTATGGTTACACTCG	GGCAGGGACAGTTGCTTCT
5	*TIMP-1*	CTTCTGCAATTCCGACCTCGT	ACGCTGGTATAAGGTGGTCTG
6	*LOX*	*CGGCGGAGGAAAACTGTCT*	*TCGGCTGGGTAAGAAATCTGA*
7	*MINCLE*	*AAGAACTGCTCAGCCATGGG*	*CCTGCTCCTCCTGTGAGTTGA*
8	*iNOS*	*TTCAGTATCACAACCTCAGCAAG*	*TGGACCTGCAAGTTAAAATCCC*
9	*TGFB1*	*GGCCAGATCCTGTCCAAGC*	*GTGGGTTTCCACCATTAGCAC*
10	*β-ACTIN*	*GTTGCTATCCAGGCTGTG*	*TGATCTTGATCTTCATTGTG*

For animal studies:

Number	Gene	Forward (5'-3')	Reverse (5'-3')
1	*Col1a1*	TGCTGGTCCCAAAGGTTC	CAGGGCGACCATCTTGAC
2	*Col3a1*	ATGCCCACAGCCTTCTACAC	ACCAGTTGGACATGATTCACAG
3	*Mincle*	ACCAAATCGCCTGCATCC	CACTTGGGAGTTTTTGAAGCATC
4	*iNOS*	CCAAGCCCTCACCTACTTCC	CTCTGAGGGCTGACACAAGG
5	*Tgfb1*	CCTGACTGGCTGTCTTTTGACG	AGTGAGCGCTGAATCGAAAGC
6	*Pdgfb*	CCATCCGCTCCTTTGATGAT	TCAGCCCCATCTTCATCTACG
7	*Acta2*	GTTCAGTGGTGCCTCTGTCA	ACTGGGACGACATGGAAAAG
8	*Il-6*	CCTTCTTGGGACTGATGCTGGTG	AGGTCTGTTGGGAGTGGTATCCTC
10	*TNF-α*	AGCCGATGGGTTGTACCTTG	GTGGGTGAGGAGCACGTAGTC
11	*β-actin*	CCACAGCTGAGAGGGAAATC	AAGGAAGGCTGGAAAAGAGC

Supplementary Figures

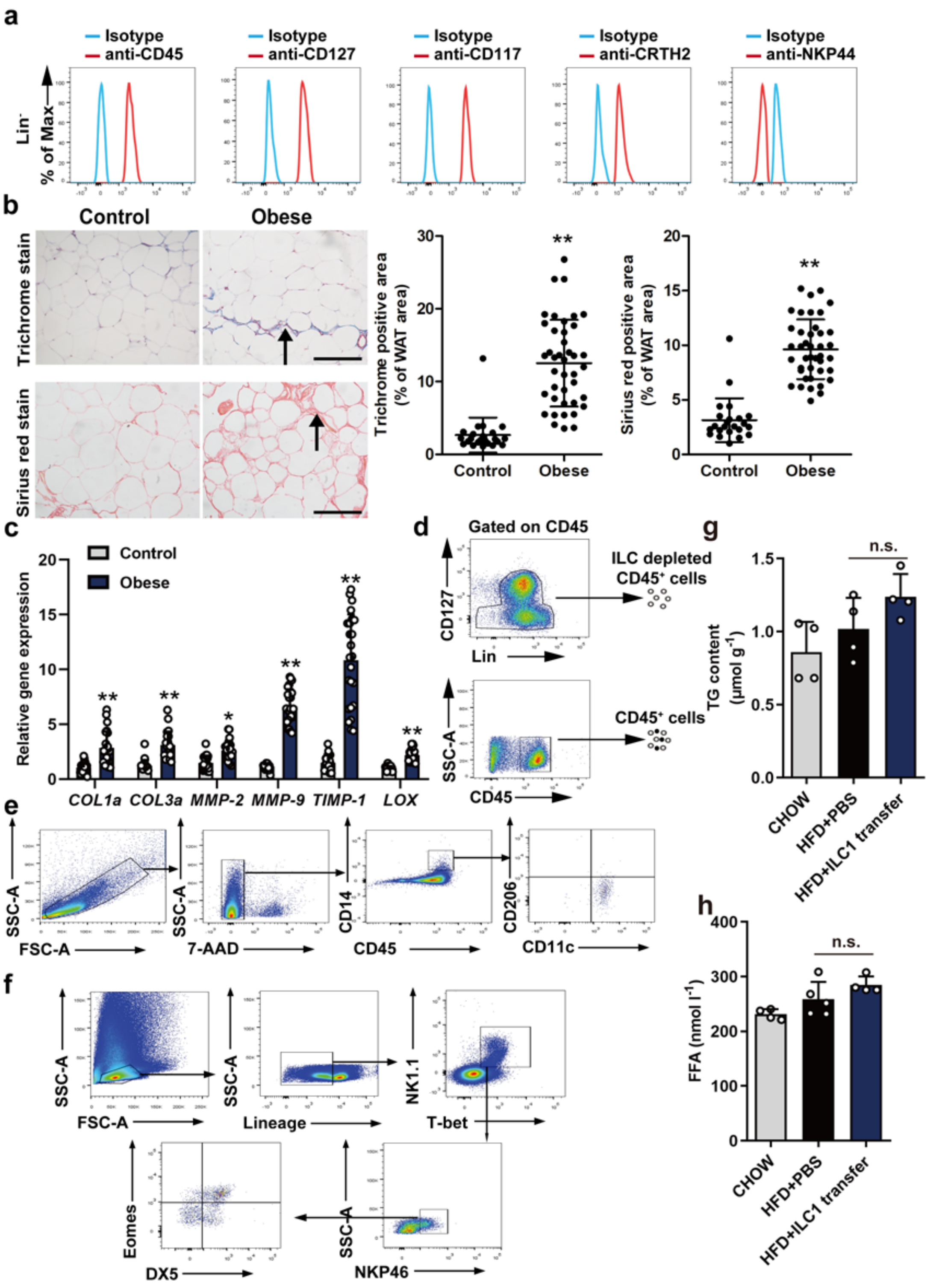

Supplementary Figure 1. a Flow cytometry analysis of cells stained with isotype controls for CD45, CD127, CD117, CRTH2, and NKP44. **b** Representative Masson's trichrome C and Sirius red staining in omental adipose tissue samples from control subjects and obese subjects (scale bar: 100μm). Quantitation of Masson's trichrome C and Sirius red staining by Image J software from NIH was performed (n=24 and 41,

respectively). ** $P<0.01$ *vs.* control group (unpaired two-way Student's *t* test). **c** Expression levels of fibrosis related genes in control subjects and obese subjects (n=24 per group). ** $P<0.01$ *vs.* control group (unpaired two-way Student's *t* test). **d** Sorting strategy by flow cytometry. **e** Analysis of macrophages in human adipose tissue by flow cytometry. **f** Representative flow cytometric analysis for adipose ILC1s in SVFs. **g,h** Hepatic TG levels and serum FFA levels. n.s., no significance (two-way ANOVA tests followed by Bonferroni post hoc test). Data are representative of three independent experiments, with n = 4-6 mice per group. Error bars indicate s.d..

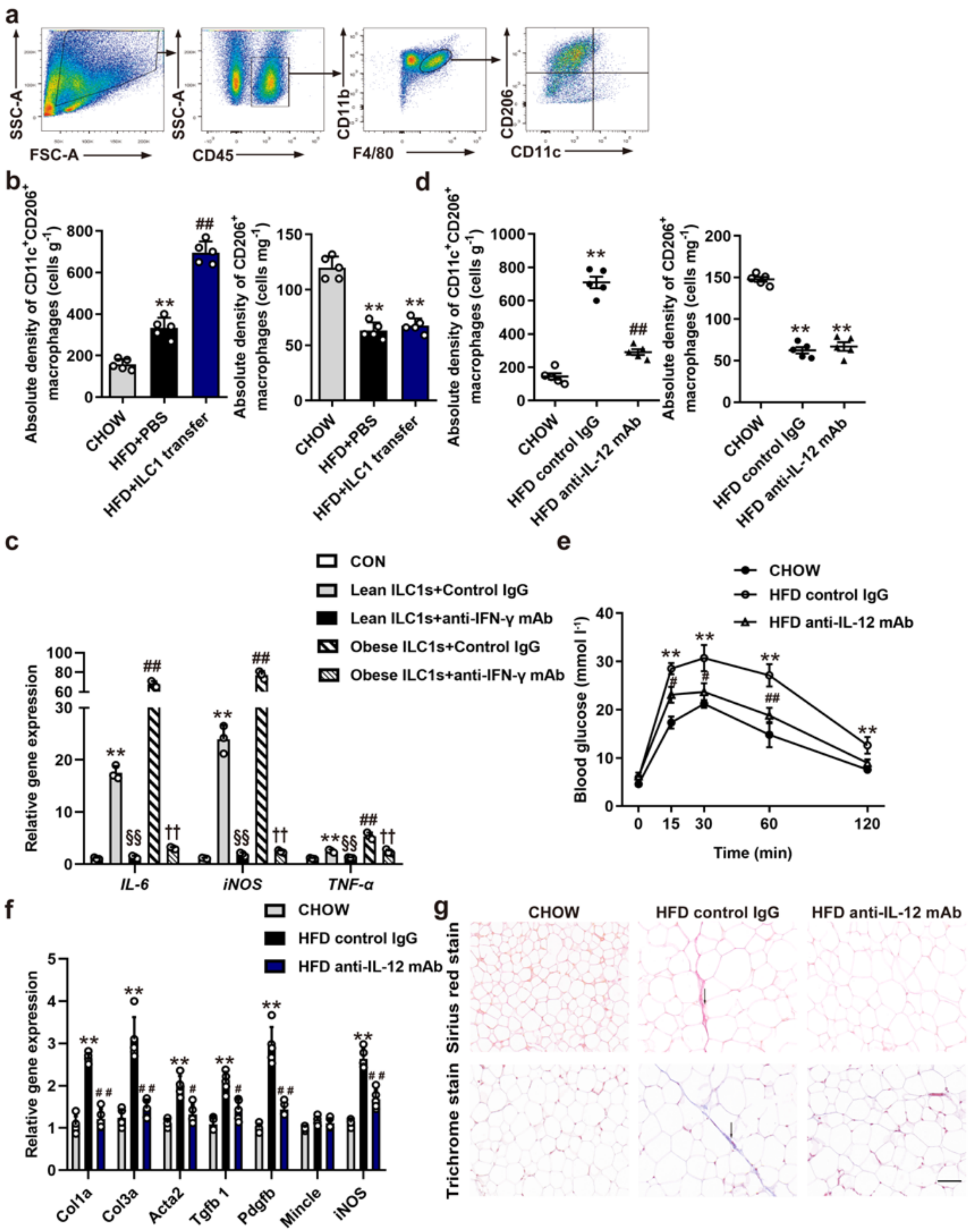

Supplementary Figure 2. a Representative flow cytometric analysis for ATMs in mice. **b** Absolute density of CD11c^{+}CD206^{+} and CD206^{+} macrophages in VAT of mice with different treatment. Data are representative of three independent experiments, with n = 4-6 mice per group. ***P*<0.01 *vs.* CHOW group; $^{\#\#}$*P*<0.01 *vs.* HFD+PBS group (two-way ANOVA tests followed by Bonferroni post hoc test). **c** Adipose ILC1s (2×10^{4} cells well^{-1}) sorted from VAT of lean and obese mice were co-cultured with BMDMs (2×10^{5} cells well^{-1}) in 24-well plates using a transwell system. Recombinant mouse IL-12 (20 ng ml^{-1}) and IL-18 (20 ng ml^{-1}) were added in the upper chamber, with neutralizing IFN-γ antibody (20 ng ml^{-1}) or isotype control IgG (20 ng ml^{-1}) supplemented in separate group. ** *P*<0.01 *vs.* control group; §§

P<0.01 *vs.* Lean ILC1s+Control IgG group; $^{\#\#}P$<0.01 *vs.* Lean ILC1s+Control IgG group; $^{\dagger\dagger}P$<0.01 *vs.* Obese ILC1s+Control IgG group (two-way ANOVA tests followed by Bonferroni post hoc test). Data are representative of three independent experiments. **d** Absolute density of $CD11c^{+}CD206^{+}$ and $CD206^{+}$ macrophages in VAT of mice with different treatment. $^{**}P$<0.01 *vs.* CHOW group; $^{\#\#}P$<0.01 *vs.* HFD+Control IgG group (two-way ANOVA tests followed by Bonferroni post hoc test). Data are representative of two independent experiments, with n = 5 mice per group. **e-g** C57BL/6 mice were fed with normal diet or HFD, while mice in the HFD group were randomized into two groups of HFD anti-IL-12 mAb group and HFD control IgG group and continued with HFD feeding for another 4 weeks. HFD anti-IL-12 mAb group received rat anti-murine anti-IL-12p35 mAb administration via intraperitoneal injection at 250 μg per mouse every 3 days for 4 weeks; HFD control IgG group received the same dose of control IgG. **(e)** Glucose tolerance test. **(f)** Expression levels of fibrosis related genes, assessed by q-PCR. **(g)** Representative Masson's trichrome C and Sirius red staining in VAT of mice with different treatment. $^{**}P$<0.01 *vs.* respective CHOW group; $^{\#}P$<0.05 *vs.* HFD+control IgG group; $^{\#\#}P$<0.01 *vs.* HFD+control IgG group (two-way ANOVA tests followed by Bonferroni post hoc test). Data are representative of two independent experiments, with n = 5-6 mice per group. Error bars indicate s.d..

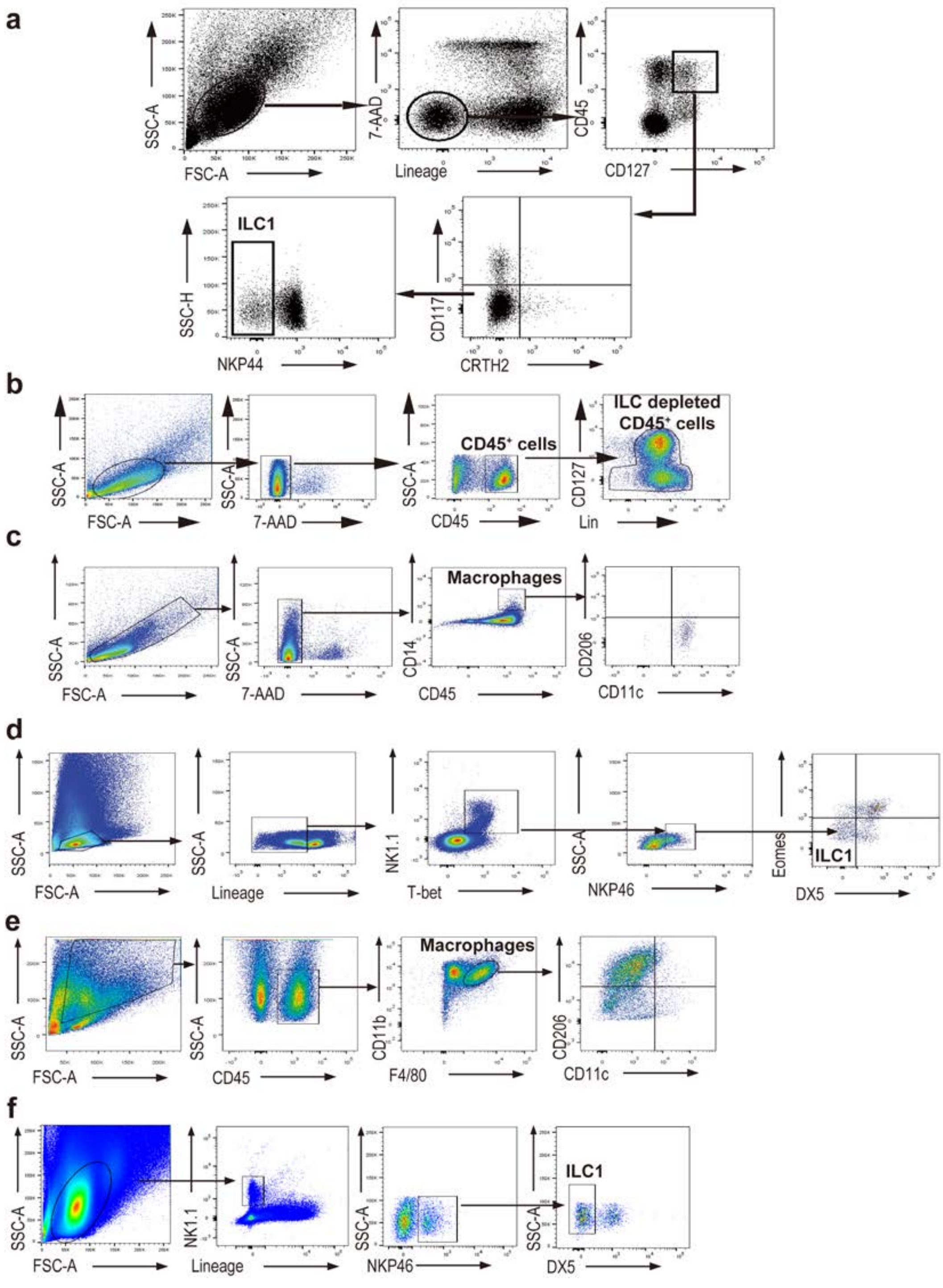

Supplementary Figure 3. Gating strategies used for cell identification and sorting. **a** Gating strategy to identify human adipose and circulating ILC1 ($Lin^-CD45^+CD127^+CD117^-CRTH2^-NKP44^-$) cells for analyses presented on Fig. 1b-k,2a. **b** Gating strategy to sort adipose $CD45^+$ cells or ILCs depleted $CD45^+$ cells from the SVFs of obese T2D patients for in-vitro co-culture assay presented on Fig. 2d,e. **c** Gating strategy to identify human adipose macrophages ($CD45^+CD14^+CD11c^+$) for analyses presented on Fig. 2d. **d** Gating strategy to identify adipose ILC1s ($Lin^-NK1.1^+NKP46^+T\text{-}bet^+Eomes^-DX5^-$) from *Prkdc*$^{-/-}$*IL2rg*$^{-/-}$ and *Rag1*$^{-/-}$ mice for analyses presented on Fig. 3b,6a. **e** Gating strategy to identify adipose ATMs

(CD45$^+$CD11b$^+$F4/80$^+$) from *Prkdc*$^{-/-}$*IL2rg*$^{-/-}$ and *Rag1*$^{-/-}$ mice for analyses presented on Fig. 4b,6c. **f** Gating strategy to sort adipose ILC1s from C57BL/6 and *Ifng*$^{-/-}$ mice for adoptive transfer assay presented on Fig. 3a,5a.

Supplementary Methods

Bone Marrow Derived Macrophages Isolation

BMDMs were isolated from C57BL/6 mice fed a normal diet as previously described[1]. In brief, bone marrow cells were flushed out from femur, resuspended and grown in DMEM/F12-10 medium (10% FBS and 1% penicillin and streptomycin) containing 10 ng ml^{-1} M-CSF (PeproTech). Fresh growth medium was added on day 3. Macrophages were harvested on day 7 and replated for further experiment.

Co-culture experiments

As for the co-culture experiment, a transwell system (BD Biosciences) was used. Adipose ILC1s were sorted from C57BL/6 mice fed a normal diet or HFD diet for 4 weeks by flow cytometry. About 2×10^5 BMDMs were seeded in 24-well plate and co-cultured with adipose ILC1s (2×10^4 cells $well^{-1}$) in the presence of neutralizing IFN-γ antibody (BioXCell, #BP0055) or IgG isotype control antibody (BioXCell, #BP0290). Meanwhile, recombinant mouse IL-12 (R&D Systems, #419-ML) and IL-18 (R&D Systems, #9139-IL) were added in the upper chamber. After co-culture for 24 hours, BMDMs were collected for further detection.

Supplementary References

1. Zhang X, Goncalves R, Mosser DM. The isolation and characterization of murine macrophages. *Current protocols in immunology* **Chapter 14**, Unit 14 11 (2008).
