# Supplementary material for: Adipose group 1 innate lymphoid cells promote adipose tissue fibrosis and diabetes in obesity
Source: Nat Commun. 2019 Jul 22;10:3254. doi: 10.1038/s41467-019-11270-1 (PMC6646407; doi:10.1038/s41467-019-11270-1)
Supplement: Supplementary file 2 — Reporting Summary [file 41467_2019_11270_MOESM2_ESM.pdf]

nature research

Corresponding author(s): Yan Bi

Last updated by author(s): Jun 13, 2019

Reporting Summary

Nature Research wishes to improve the reproducibility of the work that we publish. This form provides structure for consistency and transparency in reporting. For further information on Nature Research policies, see Authors & Referees and the Editorial Policy Checklist.

Statistics

For all statistical analyses, confirm that the following items are present in the figure legend, table legend, main text, or Methods section.

n/a	Confirmed	
☐	☒	The exact sample size (*n*) for each experimental group/condition, given as a discrete number and unit of measurement
☐	☒	A statement on whether measurements were taken from distinct samples or whether the same sample was measured repeatedly
☐	☒	The statistical test(s) used AND whether they are one- or two-sided<br>*Only common tests should be described solely by name; describe more complex techniques in the Methods section.*
☐	☒	A description of all covariates tested
☐	☒	A description of any assumptions or corrections, such as tests of normality and adjustment for multiple comparisons
☐	☒	A full description of the statistical parameters including central tendency (e.g. means) or other basic estimates (e.g. regression coefficient) AND variation (e.g. standard deviation) or associated estimates of uncertainty (e.g. confidence intervals)
☒	☐	For null hypothesis testing, the test statistic (e.g. *F*, *t*, *r*) with confidence intervals, effect sizes, degrees of freedom and *P* value noted<br>*Give P values as exact values whenever suitable.*
☒	☐	For Bayesian analysis, information on the choice of priors and Markov chain Monte Carlo settings
☒	☐	For hierarchical and complex designs, identification of the appropriate level for tests and full reporting of outcomes
☒	☐	Estimates of effect sizes (e.g. Cohen's *d*, Pearson's *r*), indicating how they were calculated

Our web collection on statistics for biologists contains articles on many of the points above.

Software and code

Policy information about availability of computer code

Data collection	No commercial source or open source data was used in this study.
Data analysis	None.

For manuscripts utilizing custom algorithms or software that are central to the research but not yet described in published literature, software must be made available to editors/reviewers. We strongly encourage code deposition in a community repository (e.g. GitHub). See the Nature Research guidelines for submitting code & software for further information.

Data

Policy information about availability of data

All manuscripts must include a data availability statement. This statement should provide the following information, where applicable:

- Accession codes, unique identifiers, or web links for publicly available datasets
- A list of figures that have associated raw data
- A description of any restrictions on data availability

The data that support the findings of this study are available from the corresponding author upon reasonable request.

Field-specific reporting

Please select the one below that is the best fit for your research. If you are not sure, read the appropriate sections before making your selection.

☒ Life sciences ☐ Behavioural & social sciences ☐ Ecological, evolutionary & environmental sciences

For a reference copy of the document with all sections, see nature.com/documents/nr-reporting-summary-flat.pdf

Life sciences study design

All studies must disclose on these points even when the disclosure is negative.

Sample size	Sample size were chosen based on prior knowledge for obtaining significant p-values from the type of experiment performed.
Data exclusions	No data were excluded.
Replication	All experiments were replicated at least twice and often more times as mentioned in the manuscript.
Randomization	Animals were randomized to diet experimental groups.
Blinding	The quantitative histological analysis was performed by two investigators who had no knowledge of the origin of the slides.

Reporting for specific materials, systems and methods

We require information from authors about some types of materials, experimental systems and methods used in many studies. Here, indicate whether each material, system or method listed is relevant to your study. If you are not sure if a list item applies to your research, read the appropriate section before selecting a response.

Materials & experimental systems

n/a	Involved in the study	
☐	☒	Antibodies
☒	☐	Eukaryotic cell lines
☒	☐	Palaeontology
☐	☒	Animals and other organisms
☐	☒	Human research participants
☐	☒	Clinical data

Methods

n/a	Involved in the study	
☒	☐	ChIP-seq
☐	☒	Flow cytometry
☒	☐	MRI-based neuroimaging

Antibodies

Antibodies used	CD3 (UCHT1), CD19 (HIB19), CD16 (B73.1), CD45 (2D1), CD127 (HIL-7R-M21), CD117 (YB5, B8), CRTH2 (BM16), NKP44 (p44-8), CD206 (19.2), CD14 (M5E2), CD11c (Bu15), CD5 (L17F12), TCRαβ (IP26), FCERIA (AER-37), TCRβ (H57-597), CD3 (17A2), CD19 (1D3), TCRγδ (GL3), Ly-6G (1A8), F4/80 (BM8), NK1.1 (PK136), NKP46 (29A1.4), DX5 (DX5), Eomes (1219A), T-bet (4B10), CD11b (M1/70), CD11c (N418), IFN-γ (4S.B3), CD206 (Co68C2) and CD45 (A20) were purchased from BioLegend, R&D Systems, or BD Biosciences. Phosphorylated Smad3 (ab52903), total Smad3 (ab40854), GAPDH (ab8245), pSTAT1 (ab109461), F4/80 (ab6640), αSMA (ab7817) and TGF-β1 antibody (ab92486) were purchased from Abcam.
Validation	We used only previously published and validated or commercial antibodies.

Animals and other organisms

Policy information about studies involving animals; ARRIVE guidelines recommended for reporting animal research

Laboratory animals	The mice used in this study were included in method and figure legend section of the manuscript. C57BL/6, Rag1-/-, Ifng-/- and Prkdc-/-IL2rg-/- mice were used.
Wild animals	The study did not involve wild mice.
Field-collected samples	The study did not involve samples collected from the field.
Ethics oversight	All animal studies were performed according to guidelines established by the Research Animal Care Committee of Drum Tower Hospital Affiliated to Nanjing University Medical School, Nanjing, China.

Note that full information on the approval of the study protocol must also be provided in the manuscript.

Human research participants

Policy information about studies involving human research participants

Population characteristics	Clinical and biochemical characteristics of the enrolled subjects are summarized in tables. Periumbilical adipose tissue samples at the omental region were obtained from 21 obese subjects, 20 obese T2D patients, and 24 control subjects perioperatively. Blood samples were collected from 19 obese patients and 17 obese T2D patients 3 months after surgery at the end of the study.

Recruitment	A total of 85 subjects, including 49 obese patients underwent laparoscopic Roux-en-Y gastric bypass (RYGB) surgery, and 36 age- and sex- matched non obese non T2D controls received elective abdominal surgery (e.g. hernia or hemangioma resection) were enrolled from April 2017 to December 2017 at Drum Tower Hospital Affiliated to Nanjing University Medical School.
Ethics oversight	This study was approved by the Ethics Review Committee of Nanjing Drum Tower Hospital Affiliated to Nanjing University Medical School (Approval number: 2017-030-02).

Note that full information on the approval of the study protocol must also be provided in the manuscript.

Clinical data

Policy information about clinical studies

All manuscripts should comply with the ICMJE guidelines for publication of clinical research and a completed CONSORT checklist must be included with all submissions.

Clinical trial registration	NCT03296605
Study protocol	*Note where the full trial protocol can be accessed OR if not available, explain why.*
Data collection	*Describe the settings and locales of data collection, noting the time periods of recruitment and data collection.*
Outcomes	*Describe how you pre-defined primary and secondary outcome measures and how you assessed these measures.*

Flow Cytometry

Plots

Confirm that:

☒ The axis labels state the marker and fluorochrome used (e.g. CD4-FITC).

☒ The axis scales are clearly visible. Include numbers along axes only for bottom left plot of group (a 'group' is an analysis of identical markers).

☒ All plots are contour plots with outliers or pseudocolor plots.

☒ A numerical value for number of cells or percentage (with statistics) is provided.

Methodology

Sample preparation	PBMCs were isolated with vacutainer cell preparation tubes (CPTs) (Becton Dickinson, Franklin Lakes, NJ) from fasting blood samples in the non-obese and obese patients pre-surgery and in the obese patients after 3 months follow-up of surgery. Periumbilical adipose tissue samples at the omental region were obtained from 21 obese subjects, 20 obese T2D patients, and 24 control subjects perioperatively. Adipose tissue samples (approximately 10 g) were collected and transported to laboratory immediately. For human SVFs preparation, fresh adipose samples were cut into small pieces and digested with 0.1% type II collagenase (Sigma-Aldrich, USA). Mouse SVFs were prepared using 0.1% type I collagenase. Intracellular cytokine staining was performed with Cytofix/Cytoperm Plus kit (BD Biosciences)
Instrument	Samples were analyzed on an LSR II Fortessa flow-cytometer (BD, Bioscience) or sorted on a FACSAria III flow-cytometer (BD Biosciences)
Software	Data were analyzed with FACSDiva software (BD, Bioscience) or Flowjo software version 9.6.4 (Tree Star, Inc.).
Cell population abundance	We provided FACS-gating strategies for all flow cytometry analysis and FACS cell sorting that confirm the abundance of the analyzed or sorted relevant cell populations.
Gating strategy	We provided FACS-gating strategies for all flow cytometry analysis and FACS cell sorting, specifying the preliminary FSC/SSC gates and how positive and negative staining cell populations are defined.

☒ Tick this box to confirm that a figure exemplifying the gating strategy is provided in the Supplementary Information.
